# Supplementary material for: Decoding a cancer-relevant splicing decision in the RON proto-oncogene using high-throughput mutagenesis
Source: Nat Commun. 2018 Aug 17;9:3315. doi: 10.1038/s41467-018-05748-7 (PMC6098099; doi:10.1038/s41467-018-05748-7)

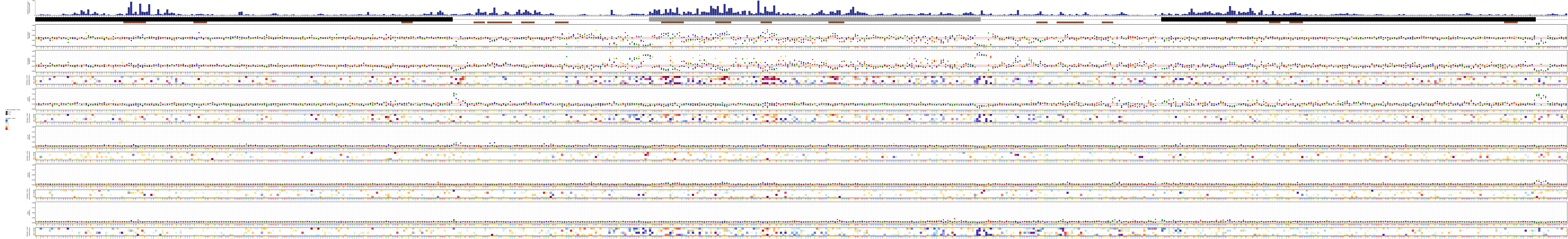

Supplement: Supplementary file 9 — Supplementary Data 6 [file 41467_2018_5748_MOESM9_ESM.pdf]
