# Supplementary material for: Decoding a cancer-relevant splicing decision in the RON proto-oncogene using high-throughput mutagenesis
Source: Nat Commun. 2018 Aug 17;9:3315. doi: 10.1038/s41467-018-05748-7 (PMC6098099; doi:10.1038/s41467-018-05748-7)
Supplement: Supplementary file 1 — Supplementary Information [file 41467_2018_5748_MOESM1_ESM.pdf]

Decoding a cancer-relevant splicing decision in the *RON* proto-oncogene using high-throughput mutagenesis

SUPPLEMENTARY INFORMATION

Braun et al.

<u>Content</u>:

Supplementary Notes **2**
Supplementary Note 1: Dynamic model of splicing reactions **2**
1.1 *In silico* simulation of competing splicing reactions 3
Supplementary Note 2: Inference of single mutation effects **4**
2.1 Calculation of single mutation effects by linear regression 4
2.2 Comparative analysis of linear regression approaches 6
2.3 Estimation of the prediction error of the model 7
Supplementary Note 3: Model analysis of *HNRNPH* knockdown effects **10**
Supplementary Tables 1 - 4 .... **12**
Supplementary Figures 1 - 16 .... **19**
Supplementary References **37**

Supplementary Notes

In these Supplementary Notes, we describe how we used mathematical modelling to infer the effect of single mutations on the splicing outcome. We employed a two-step modelling strategy in which we first calculate changes in splicing reactions from the isoform frequency using a dynamical model (Supplementary Note 1). In the second step, we describe the splice change in each minigene variant harbouring multiple mutations as a linear combination of single mutation effects, and estimate these single effects using a regression approach (Supplementary Note 2). Finally, we compare single mutation effects for control and *HNRNPH* knockdown conditions to identify synergistic interactions between these two types of perturbations (Supplementary Note 3).

Supplementary Note 1: Dynamic model of splicing reactions

We modelled the dynamics of splicing using a set of ordinary differential equations, in which concentrations of transcript intermediates are determined by production and degradation terms. The precursor mRNA (pre-mRNA) x_0 is produced at a constant rate c and spliced into different splice products with linear kinetics and rates r_i, leading to

$$\frac{dx_0}{dt} = \mathrm{c} - (r_1 + r_2 + r_3 + r_4 + r_5 + r_6)x_0. \qquad (1)$$

Additional differential equations describe the dynamics of the spliced isoforms:

$$\frac{dx_i}{dt} = r_i x_0 - d_i x_i, i = 1, \ldots, 6. \qquad (2)$$

where $x_1 \ldots, x_5$ are the number of transcripts representing the alternative exon (AE) inclusion, AE skipping, full intron retention (IR), first IR and second IR isoforms. The additional non-canonical isoforms that were also measured are integrated in the model together by the species x_6, collectively referred to as 'other'. Furthermore, d_i are the degradation rates of the different isoforms.

The steady state found by setting dx_i/dt to zero in Supplementary Equations 2 reads $x_i = (r_i/d_i)x_0$. The measured isoform frequencies p_i correspond in the model to the fractions of the transcripts x_i within the total mRNA:

$$p_i = \frac{x_i}{x_0+x_1+x_2+x_3+x_4+x_5+x_6}, i = 1,2,4,5,6. \qquad (3)$$

For the frequency p_3 of mRNA transcripts that exhibit the complete sequence, we sum up the number of mRNA transcripts with full intron retention x_3 and the number of unspliced pre-mRNA transcripts x_0, since these two species were experimentally not differentiated. Thus, we get

$$p_3 = \frac{x_0+x_3}{x_0+x_1+x_2+x_3+x_4+x_5+x_6}. \qquad (4)$$

At steady state, we obtain from Supplementary Equations 1-4

$$p_i = \frac{K_i}{K_1+K_2+K_3+K_4+K_5+K_6}, i = 1, \ldots, 6. \qquad (5)$$

where we introduced the parameters $K_i = r_i/d_i, i = 1,2,4,5,6$ for the isoforms involving splicing and $K_3 = 1 + r_3/d_3$ for the unspliced full IR isoform.

We remark that due to the normalisation condition $\sum_{i=1}^{6} p_i = 1$, not all model parameters K_i can be determined from the experimental data, but only ratios of K_i with respect to a reference isoform. If we normalise all K_i by the AE inclusion rate, we can determine the ratios $K_i/K_1, i = 2, \ldots, 6$ from the measured isoform frequencies via $K_i/K_1 = p_i/p_1$.

1.1 *In silico* simulation of competing splicing reactions

Supplementary Equation 5 reflects the non-linear nature of the splicing system: For example, a perturbation affecting the splicing parameter K_2 will affect all transcript isoform frequencies $p_i$if $K_2$is large compared to other parameters, but not otherwise. In contrast, the splice isoform ratios respond in the same way to a perturbation affecting the splicing parameter K_2, irrespective of the other parameter values.

We confirmed that perturbation-induced fold-changes in the isoform frequencies, but not isoform ratios, depend on the mutational background by numerically simulating the steady state of the splicing system (Supplementary Equations 1 and 2). Random mutagenesis (i.e., the varying mutational background) was mimicked by uniformly sampling parameters $\mathrm{c}, r_i, d_i, i = 1, \ldots, 6$ in logarithmic space within the range [0.1,10], and calculating the steady state for 5,000 different realisations. Subsequently, each parameter set was additionally perturbed by decreasing the parameter r_2 at 20% of the sampled value (representing an additional mutation or knockdown), and the new steady state was calculated. As expected from the inspection of the steady states given in Supplementary Equation 5, the effect of the perturbation on splice isoforms frequencies is nonlinear and strongly depends on the specific parameter values, i.e., the mutational background (**Supplementary Fig. 7e**).

In contrast, the perturbation of r_2 has a linear effect on the splice isoform ratios in the sense that the fold-change between perturbed and unperturbed steady states is the same for all parameter sets (**Supplementary Fig. 7e**). Therefore, a mutation (or knockdown) affecting splicing kinetics induces the same fold change of an isoform ratio, irrespective of the presence of other mutations in the minigene. Thus, perturbation effects on splice isoform ratios show additive behaviour in log-space and are therefore more suitable for the regression approach described below.

Supplementary Note 2: Inference of single mutation effects

2.1 Calculation of single mutation effects by linear regression

By analysing the cumulative mutational effects in minigenes containing two or three mutations that are also present as single mutations in other minigenes, we found that the effects of single mutations on the above defined splicing rates are in general multiplicative (**Supplementary Fig. 4a**). Thus, we assume that the splicing parameter K_i for a minigene exhibiting a combination of several mutations is given by

$$K_i^{\text{mutated}} = K_i^{\text{wild type}} m_i^1 m_i^2 \dots m_i^n, \quad (6)$$

where n is the number of the mutations in the minigene and m_i^k the effect of the k-th mutation on K_i.

Using the same normalisation to the AE inclusion isoform as in Supplementary Note 1, and taking the logarithm of Supplementary Equation 6 leads to

$$\sum_{k=1}^{n} \log \frac{m_i^k}{m_1^k} = \log \frac{p_i}{p_1} - \log \frac{p_i^{\text{wt}}}{p_1^{\text{wt}}}, i = 2, \dots, 6, \quad (7)$$

where p_i and $p_i^{\text{wt}}, i = 1, \dots, 6$ are the isoform frequencies of the mutated and wild type (wt) *RON* minigenes, respectively. The isoform frequencies for the wt *RON* minigene were calculated as the median of the measured values across the minigenes exhibiting the wt sequence (586 minigene variants present in all RNA-seq replicates).

By considering all minigene variants together, we get a system of linear equations for the mutational effects $x_i(k) = \log\left(m_i^k / m_1^k\right), i = 2, \dots, 6, k = 1, \dots, N$, where N is the total number of mutations present in the dataset. For each of the five splice isoform ratios K_i/K_1, we get a separate system of linear equations which can be written in the matrix form:

$$A\mathbf{x}_i = \mathbf{b}_i, i = 2, \dots, 6. \quad (8)$$

The entries of the matrix are $A(j,k) = 1/0$ if mutation k is present/absent in minigene variant j, respectively. The vectors $\mathbf{b}_i$ contain the experimental observations which are given by

$$b_i(j) = \log \frac{p_i^j}{p_1^j} - \log \frac{p_i^{\text{wt}}}{p_1^{\text{wt}}}, i = 2, \dots, 6, j = 1, \dots, m, \quad (9)$$

with m being the number of unique combinations of mutations included in the calculation (between 4,467 and 4,771, depending on the cell line and replicate, see **Supplementary Table 1**).

Since any minigene contains only a few of the total unique 2,042 mutations present in the whole dataset (up to 18 mutations, with a mean of 3.7 mutations/minigene including insertions and deletions), the systems to be solved are sparse. To get the single mutational effects $x_i(k)$, we solved the systems in Supplementary Equations 8 in least square sense using Matlab subroutine lscov.

From the estimated mutational effects $x_i(k)$, a model prediction for the isoform frequencies p_i^k in a minigene containing the single mutation k can be made: For the single-mutation minigene, we would have

$$\frac{p_i^k}{p_1^k} = \frac{p_i^{\text{wt}}}{p_1^{\text{wt}}} e^{x_i(k)}, i = 2, \dots, 6. \quad (10)$$

By summing up Supplementary Equations 10 and using the normalisation condition $\sum_{i=1}^{6} p_i^k = 1$, we therefore get

$$\frac{1-p_1^k}{p_1^k} = \sum_{i=2}^{6} \frac{p_i^{\mathrm{wt}}}{p_1^{\mathrm{wt}}} \mathrm{e}^{x_i(k)}, \qquad (11)$$

which can be solved to find the AE inclusion isoform frequency p_1^k as a function of the single mutation effects:

$$p_1^k = \frac{1}{1+\sum_{i=2}^{6} \frac{p_i^{\mathrm{wt}}}{p_1^{\mathrm{wt}}} \mathrm{e}^{x_i(k)}} = \frac{p_1^{\mathrm{wt}}}{p_1^{\mathrm{wt}}+\sum_{i=2}^{6} p_i^{\mathrm{wt}} \mathrm{e}^{x_i(k)}}. \qquad (12)$$

Finally, the remaining isoform frequencies can be estimated via:

$$p_i^k = p_1^k \frac{p_i^{\mathrm{wt}}}{p_1^{\mathrm{wt}}} \mathrm{e}^{x_i(k)} = \frac{p_i^{\mathrm{wt}} \mathrm{e}^{x_i(k)}}{p_1^{\mathrm{wt}}+\sum_{i=2}^{6} p_i^{\mathrm{wt}} \mathrm{e}^{x_i(k)}}, i = 2,\ldots,6. \quad (13)$$

Supplementary Equations 8 and 9 were used to infer the effects of single mutations from the data. Different replicates were treated separately, since both wt and mutated minigene variants showed systematic shifts in the measured frequencies between replicates. Thus, we always calculate mutational effects by comparing isoform frequencies of mutated and wt minigenes within the same replicate.

We note that the library also contains some minigenes with different barcodes but the same combination of mutations. We have included such combinations of mutations only once and attributed to them the median of the measured isoforms frequencies over the different minigenes with the same combination of mutations. Thus, the number of unique combinations of mutations is smaller than the number of mutated minigene variants (i.e. unique barcodes) in the dataset (**Supplementary Table 1**). Furthermore, we have excluded barcodes containing ambiguous mutations from the calculation.

The predictive power of our modelling approach was confirmed using cross-validation (also see Methods; **Supplementary Fig. 6**), and by comparing the inferred splicing outcome in response to single mutations (according to Supplementary Equations 11 and 12) to RT-PCR measurements of previously untested minigenes containing only these single mutations (see main manuscript; **Fig. 2d**).

It should be noted that certain minigenes had to be excluded from the linear regression procedure because they deviated from linear behaviour: (i) Minigenes simultaneously harbouring two splice site mutations: these minigenes show a very similar distribution of inclusion frequencies as minigenes containing only one of these mutations (**Supplementary Fig. 7f**). The median inclusion frequency of both, one- and two-splice-site-mutation minigenes, was non-zero (0.7%). The apparent lack-of-effect of secondary splice site mutations at non-zero inclusion frequencies contradicts their strong effect as isolated splice site mutations, and introduces strong inconsistencies and biases in linear regression. In our opinion, this observation hints to a constant background signal, e.g., due to leaky sequencing reads originating from other minigenes where inclusion is the predominant isoform. Therefore, we excluded minigenes exhibiting any two mutations at positions proximal to splice sites (positions 210-212, 295-297, 443-446, 522-524, 689-691). (ii) Minigenes with strong activation of cryptic splice sites: The activation of cryptic splice sites by mutations leads to the generation of a plethora of new splicing products ('other') which behave heterogeneously and cannot be considered in our model. Therefore, we performed first the regression on the complete dataset and subsequently excluded the minigenes containing

mutations that were predicted to exhibit an increased 'other' isoform frequency $p_6 > 4p_6^{\mathrm{wt}}$ in this first run. The threshold used for the exclusion of minigenes from the regression dataset was four times the median p_6^{wt} of the 'other' isoform frequency for the wt minigenes, and thus cell line and replicate-specific. The final calculation of mutational effects was performed on this reduced dataset (**Supplementary Table 1**). As an alternative approach to estimate the mutation effects of the excluded mutations, we calculated the median of isoform frequencies for all minigene variants harbouring the given mutation (**Supplementary Data 3**).

Depending on the replicate and cell line, between 3-9% of the unique combinations of mutations were excluded from the calculations based on the above criteria (**Supplementary Table 1**). Still, the effects of 94-97% of the mutations present in the library could be assessed by regression that covered almost the entire length of the minigene (all but 3-4 out of all 679 nucleotides in the minigene).

2.2 Comparative analysis of linear regression approaches

As described above, kinetic modelling suggested that fitting to splice isoform ratios is most suitable for linear regression. To support this claim, we tested two alternative regression approaches for the inference of single mutation effects, both of which were based on direct fitting to splice isoform frequencies. Reassuringly, our isoform ratio-based approach outperformed these alternative methods.

First, we assumed that the mutation effects add up at the level of splice isoform frequencies (not at the level of ratios). Thus, we used

$$\sum_{k=1}^{n} \log m_i^k = \log p_i - \log p_i^{\mathrm{wt}}, i = 1, \ldots, 6 \qquad (14)$$

instead of Supplementary Equation 7 for the computation of the single mutation effects m_i^k. The corresponding isoform frequencies for the single mutation minigene k then read

$$p_i^k = p_i^{\mathrm{wt}} m_i^k, i = 1, \ldots, 6. \qquad (15)$$

Supplementary Equation 14 was solved in least square sense using the Matlab subroutine fmincon with the constraint that all isoform frequencies in a single mutation background are bounded to unity, i.e., $\sum_{i=1}^{6} p_i^k = 1, k = 1, \ldots, N$. During cross-validation, predictions for new combinations of mutations were given by

$$p_i = p_i^{\mathrm{wt}} m_i^1 m_i^2 \ldots m_i^n, i = 1, \ldots, 6, \qquad (16)$$

where $m_i^1, \ldots, m_i^n$ are the infered single mutation effects, and $1, \ldots, n$ the mutations present in the new combined minigene. We have compared the prediction performance of this method to the isoform ratio-based regression in 10-fold cross-validation and found that the use of isoform frequencies instead of ratios is inferior in terms of the prediction-data correlation. The corresponding Pearson correlation coefficients between model-predicted isoform frequencies and measured values for each predicted subset not used in fitting are visualised in **Supplementary Fig. 7b**. The predictions of the frequency-based model were in many cases also qualitatively wrong, as isoform frequencies of minigenes were not bounded to 1, thus leading to mispredictions $p_i > 1$, especially for the AE skipping isoform. In contrast, the calculation of isoform frequencies by renormalisation of the ratio-based regression results (Supplementary Equations 12 and 13) inherently prevents such biologically unreasonable mispredictions.

As a second alternative approach, we used multinomial logistic regression to infer the isoform frequencies in single-mutation minigenes. In this case, the dataset was categorised by introducing

six copies of each minigene and assuming as splicing output a different isoform for each of the copies. The data was weighted by the measured isoform frequencies, so each of the six samples corresponding to one minigene got as weight the measured frequency of its output isoform. We used the Python package scikit-learn with cross entropy loss and L2 regularisation to infer the probabilities for each splicing isoform for single-mutation minigenes and minigenes with new combinations of mutations. The prediction performance of this method in 10-fold cross-validation was also inferior to the isoform ratios-based regression, as shown in **Supplementary Fig. 7a**.

2.3 Estimation of the prediction error of the model

The prediction accuracy for a single mutation effect depends on the occurrence of the mutation in the minigene library. To quantitatively benchmark the accuracy of our model, we focused on ~600 mutations whose effects have been measured directly in our dataset as minigenes containing single mutations.

Benchmarking was done by eliminating the corresponding single-mutation minigenes from the dataset (separately for each of these mutations) and repeating the linear regression for the remaining data, or after removing further minigenes containing this mutation. This procedure allowed us to estimate how the prediction error depends on the occurrence of a mutation in the minigene library.

After calculating the single mutation effects, the isoform frequencies were estimated (Supplementary Equations 12 and 13) and the values for the mutations of interest were compared to the measured isoform frequencies of the single-mutation minigene. We find that the standard deviation of the prediction error (over all mutations and permutations) decreases with the occurrence of the mutation in the subset used in linear regression by $1/\sqrt{\mathrm{occurrence}}$ (see main manuscript; **Fig. 2c**).

This relationship can also be proven analytically by exploiting the profile likelihood which characterises the measurement-compliant range for each parameter value in the model (Raue et al., 2009). The agreement of the experimental data $\mathbf{b}_i$ with the model simulations $\mathbf{x}_i$ is measured by the sum of squared residuals:

$$\chi_i^2(\mathbf{x}_i) = \|A\mathbf{x}_i - \mathbf{b}_i\|^2 = \sum_{j=1}^{m}[\sum_{k=1}^{N} A\,(j,k)x_i(k) - b_i(j)]^2. \qquad (17)$$

The optimal values $\widehat{\mathbf{x}_i}$ of the model parameters estimated by linear regression minimise the objective functions χ_i^2, thus we have

$$\boldsymbol{\nabla}\chi_i^2(\widehat{\mathbf{x}_i}) = 2(A\mathbf{x}_i - \mathbf{b}_i)^{\mathrm{T}}A = 0. \qquad (18)$$

The confidence interval for a certain parameter $x_i(k)$ can be derived from the curvature of the objective functions, for example by calculating the Hessian matrices $H_i = \boldsymbol{\nabla}^{\mathrm{T}}\boldsymbol{\nabla}\chi_i^2(\widehat{\mathbf{x}_i})$. We find

$$H_i = 2A^{\mathrm{T}}A. \qquad (19)$$

The matrix A indicates the presence/absence of a particular mutation in a particular minigene variant, i.e. $A(j,k) = 1$ if mutation k is found in minigene variant j and $A(j,k) = 0$ otherwise. We therefore get for the diagonal elements of $A^{\mathrm{T}}A$

$$(A^{\mathrm{T}}A)_{kk} = \sum_{j=1}^{m} A\,(j,k)A(j,k) = \mathrm{occurrence}(k). \qquad (20)$$

which is equal to the number of minigene variants that exhibit the mutation k. For the non-diagonal elements of $A^{\mathrm{T}}A$, we get

$(A^T A)_{kl} = \sum_{j=1}^{m} A(j,k)A(j,l) = \text{occurrence}(k,l), k \neq l,$ (21)

which is equal to the number of minigenes that simultaneously exhibit the mutations k and l.

Therefore, the Taylor expansion of the objective function χ_i^2 around the minimum $\chi_i^2(\hat{\mathbf{x}}_i)$ is up to the second order given by

$$\chi_i^2(\mathbf{x}_i) = \chi_i^2(\hat{\mathbf{x}}_i) + \sum_{k=1}^{N} \text{occurrence}\,(k)[x_i(k) - \hat{x}_i(k)]^2 + \sum_{k=1}^{N} \sum_{l=1,l\neq k}^{N} \text{occurrence}\,(k,l)[x_i(k) - \hat{x}_i(k)][x_i(l) - \hat{x}_i(l)]. \quad (22)$$

Supplementary Equation 22 can be used to find the confidence intervals for the model parameters calculated by regression. For a given value of the parameter $x_i(k_0) = \hat{x}_i(k_0) + \delta_0$, the remaining parameters $x_i(k \neq k_0)$ can be refitted. Introducing $x_i(k) = \hat{x}_i(k) + \delta_k, k \neq k_0$ and using Supplementary Equation 18 for $k \neq k_0$ leads to a reduced system of equations for $\delta_{k\neq k_0}$, that can be written in matrix form as

$$C_{k_0}\left(\delta_{1,}\ldots,\delta_{k_0-1},\delta_{k_0+1},\ldots,\delta_N\right)^{\mathrm{T}} = -\mathbf{c}_{k_0}^{\mathrm{T}}\delta_0. \quad (23)$$

Thereby, the symmetric matrix C_{k_0} is found by deleting the k_0th row and column from $A^{\mathrm{T}}A$, thus

$$C_{k_0} = (A^{\mathrm{T}}A)(k,l), k \neq k_{0,} l \neq k_0. \quad (24)$$

Furthermore, the vector $\mathbf{c}_{k_0}$ contains the nondiagonal elements of the k_0th row of $A^{\mathrm{T}}A$:

$$\mathbf{c}_{k_0} = [\text{occurrence}(k_0,1),\ldots.,\text{occurence}(k_0,k_0-1),\text{occurrence}(k_0,k_0+1),\ldots.,\text{occurrence}(k_0,N)]^T. \quad (25)$$

Solving Supplementary Equation 23 leads to the optimal values for the parameters $\delta_{k\neq k_0}$:

$$\left(\delta_{1,}\ldots,\delta_{k_0-1},\delta_{k_0+1},\ldots,\delta_N\right)^{\mathrm{T}} = -C_{k_0}^{-1}\mathbf{c}_{k_0}\delta_0. \quad (26)$$

Introducing these solutions in Supplementary Equation 22 and regrouping the terms gives us

$$\chi_i^2(\delta_0) = \chi_i^2(\hat{\mathbf{x}}_i) + \text{occurrence}(k_0)\delta_0^2 + \sum_{k=1,k\neq k_0}^{N} \text{occurrence}\,(k_0,k)\delta_0\delta_k + \sum_{k=1,k\neq k_0}^{N} \sum_{l=1,l\neq k_0}^{N} \text{occurrence}\,(k,l)\delta_l\delta_k. \quad (27)$$

By using the above notations in Supplementary Equations 23 and 25 as well as Supplementary Equation 26, we find

$$\sum_{k=1,k\neq k_0}^{N} \text{occurrence}\,(k_0,k)\delta_0\delta_k = \mathbf{c}_{k_0}^{\mathrm{T}}\delta_0\left[-C_{k_0}^{-1}\mathbf{c}_{k_0}\delta_0\right] = -\mathbf{c}_{k_0}^{\mathrm{T}}C_{k_0}^{-1}\mathbf{c}_{k_0}\delta_0^2 \quad (28)$$

and

$$\sum_{k=1,k\neq k_0}^{N} \sum_{l=1,l\neq k_0}^{N} \text{occurrence}\,(k,l)\delta_l\delta_k = \left(\delta_{k\neq k_0}\right)^{\mathrm{T}} C_{k_0}\left(\delta_{k\neq k_0}\right) = \left[C_{k_0}^{-1}\mathbf{c}_{k_0}\right]^T C_{k_0}C_{k_0}^{-1}\mathbf{c}_{k_0}\delta_0^2 = \mathbf{c}_{k_0}^{\mathrm{T}}C_{k_0}^{-1}\mathbf{c}_{k_0}\delta_0^2. \quad (29)$$

where we used the symmetry $C_{k_0}^{\mathrm{T}} = C_{k_0}$. Introducing Supplementary Equations 28 and 29 in Supplementary Equation 27 finally gives us the variation of the objective function with δ_0:

$$\chi_i^2(\delta_0) = \chi_i^2(\hat{\mathbf{x}}_i) + \text{occurrence}(k_0)\delta_0^2. \quad (30)$$

Supplementary Equation 30 defines a parable with the minimal value $\chi^2(\hat{\mathbf{x}}_i)$ having the curvature $2\text{occurrence}(k_0)$. Thus, the more frequent the mutation k_0 is in the dataset, the steeper is the

parable and more constrained is the model parameter corresponding to this mutation. Setting a confidence threshold th for the objective function, e.g. imposing $\chi_i^2(\mathbf{x}_i) < \chi_i^2(\hat{\mathbf{x}}_i) + \mathrm{th}$, defines a confidence interval with respect to variation of the parameter $x_i(k_0)$ given by

$$|x_i(k_0) - \hat{x}_i(k_0)| < \sqrt{\frac{\mathrm{th}}{\mathrm{occurrence}(k_0)}}, \quad (31)$$

which confirms the result obtained numerically by validation with the single-mutation minigenes (see main manuscript; **Fig. 2c**).

Supplementary Note 3: Model analysis of *HNRNPH* knockdown effects

We compared the effect of *HNRNPH* knockdown (KD) on wt and mutant minigene variants to identify synergistic interactions between both types of perturbations that may hint to the strengthening or weakening of HNRNPH binding sites by mutations (**Fig. 5a**). Using linear regression, we sought to trace back these synergistic interactions between mutations and *HNRNPH* KD to the single mutation level.

We initially checked the validity of our splice rate model (**Supplementary Fig. 3a**; see Supplementary Note 1) for the *HNRNPH* KD data: In the primary data, the fold-change in each isoform frequency upon *HNRNPH* KD is not stable and depends on the baseline value of the mutated minigene variant under non-targeting control conditions (**Supplementary Fig. 13a**). This can be understood from Supplementary Equation 5, in which a KD affecting a splice rate K_i has a strong (linear) effect or a weak (less than linear) effect depending on how K_i relates to the other competing splice rates $K_{j\neq i}$. To correct for this effect and to facilitate linear regression modelling, we therefore employed ratios of splice isoform frequencies, which show a similar effect (fold-change) of the *HNRNPH* KD for the majority of minigenes (**Fig. 5b**, right, and **Supplementary Fig. 13b**). This can be explained as follows: If for all minigenes, the splice parameters in the *HNRNPH* KD $\bar{K}_\iota$ relate to the control splice parameters K_i by the same, isoform and KD-specific factors α_i

$$\bar{K}_\iota = \alpha_i K_i, i = 1,\ldots,6, \qquad (32)$$

then the isoform ratios $\bar{p}_\iota/\bar{p}_1$ and p_i/p_1 in *HNRNPH* KD and control conditions will also be related by the factors α_i/α_1, independent of splice-rate competition effects. This suggests that the splice model is able to correct for nonlinearities in the data, thereby facilitating the identification of true synergistic interactions.

Large discrepancies from the linear behaviour in Supplementary Equation 32 imply that a particular minigene variant reacts differently than the majority of the library to the *HNRNPH* KD, pointing to a change in a binding site of HNRNPH itself or other means that enhance or repress its function (positive or negative synergy). We used modelling to identify such synergistic interactions of sequence mutations and *HNRNPH* KD at single-nucleotide resolution. Instead of calculating KD-induced fold-changes per minigene, we employed linear regression modelling to infer single mutation effects before comparing KD effects on wt minigenes and individual mutations.

By the linear regression setup (see Supplementary Note 2), we can determine the mutational effects of single mutations in control $x_i(k)$ and KD $\bar{x}_\iota(k)$ conditions. According to our model, we have

$$\frac{\bar{K}_i^k}{\bar{K}_1^k} = \frac{\bar{K}_i^{\mathrm{wt}}}{\bar{K}_1^{\mathrm{wt}}} \mathrm{e}^{\bar{x}_\iota(k)}, \frac{K_i^k}{K_1^k} = \frac{K_i^{\mathrm{wt}}}{K_1^{\mathrm{wt}}} \mathrm{e}^{x_i(k)}, i = 2,\ldots 6, k = 1,\ldots,N. \qquad (33)$$

Using Supplementary Equation and assuming the same KD factors α_i on both mutated and wt minigenes, we get

$$\frac{\bar{K}_i^k}{\bar{K}_1^k} = \frac{\alpha_i}{\alpha_1} \frac{K_i^k}{K_1^k}, \frac{\bar{K}_i^{\mathrm{wt}}}{\bar{K}_1^{\mathrm{wt}}} = \frac{\alpha_i}{\alpha_1} \frac{K_i^{\mathrm{wt}}}{K_1^{\mathrm{wt}}}, i = 2,\ldots 6, k = 1,\ldots,N. \qquad (34)$$

From Supplementary Equations 33 and 34, we find

$$\frac{\bar{K}_i^k}{\bar{K}_1^k} = \frac{\bar{K}_i^{\mathrm{wt}}}{\bar{K}_1^{\mathrm{wt}}} \mathrm{e}^{\bar{x}_\iota(k)} = \frac{\alpha_i}{\alpha_1} \frac{K_i^{\mathrm{wt}}}{K_1^{\mathrm{wt}}} \mathrm{e}^{\bar{x}_\iota(k)}, \qquad (35)$$

and

$$\frac{\bar{K}_i^k}{\bar{K}_1^k} = \frac{\alpha_i}{\alpha_1}\frac{K_i^k}{K_1^k} = \frac{\alpha_i}{\alpha_1}\frac{K_i^{\mathrm{wt}}}{K_1^{\mathrm{wt}}}\mathrm{e}^{x_i(k)}. \qquad (36)$$

By comparing Supplementary Equations 35 and 36 we conclude that the mutation effects $x_i(k)$ should not change significantly between control and KD conditions, e.g.

$$\bar{x}_i(k) = x_i(k) \qquad (37)$$

should be valid for all mutations present in minigenes that react to the *HNRNPH* KD similarly to the wt minigenes. By contrast, above-average deviations from Supplementary Equation 37 are expected for mutations present in minigenes that react non-linearly to the KD.

We used *z*-scores to quantify to what extent a mutation shows different effects under control and *HNRNPH* KD conditions:

$$z_i^{\mathrm{kd}}(k) = \frac{x_i(k) - \bar{x}_i(k)}{\delta_i^{\mathrm{wt}}}, i = 2, \ldots, 6, k = 1, \ldots, N. \qquad (38)$$

Due to the additivity of perturbation effects, this *z*-score can be interpreted to reflect differential *HNRNPH* KD effects in wt vs. single mutant backgrounds, allowing us to formulate positive and negative synergy as stronger or weaker KD responses in mutants compared to wt (see **Fig. 5b** and main text). In these *z*-scores, the difference between *HNRNPH* KD and control behaviour is normalised by the variation of KD effects in the wt minigenes to correct for experimental noise: Based on the wt minigenes present in both control and KD datasets, the standard deviation for the wt difference between control and KD conditions can be calculated by

$$\delta_i^{\mathrm{wt}} = \mathrm{STD}\left\{\log\frac{p_i^{\mathrm{wt}}}{p_1^{\mathrm{wt}}} - \log\frac{\bar{p}_i^{\mathrm{wt}}}{\bar{p}_1^{\mathrm{wt}}}\right\}, i = 2, \ldots 6. \qquad (39)$$

When calculating synergies between mutations and knockdowns using *z*-scores, the results may become unstable if one of the two perturbations already induces a close-to-maximal effect on the splice isoform frequencies. In fact, when analysing the variation of *z*-scores over the three replicates, we find that mutations that shift the inclusion frequency close to 0% increase the error in synergy *z*-score calculations and are thus potentially problematic. We show this effect in **Supplementary Fig. 7g**, in which we plot the uncertainty of the synergy *z*-score (standard deviation over the three replicates) against the (inferred) inclusion frequency in a single-mutation minigene.

Supplementary Tables 1 - 4

Supplementary Table 1: Information on the input and output data of the mathematical model on the different RNA-seq replicates.

	**HEK293T**			**MCF7 – control**			**MCF7 – *HNRNPH* KD**		
	Rep 1	Rep 2	Rep 3	Rep 1	Rep 2	Rep 3	Rep 1	Rep 2	Rep 3
**General information**									
Internal ID	imb_koenig_2015_13			imb_koenig_2016_07			imb_koenig_2016_08		
Initial reads	17,261,922	19,501,750	18,166,077	19,103,473	17,132,590	22,075,639	17,956,862	19,551,048	21,930,173
Minigenes	5,697	5,645	5,623	5,680	5,680	5,684	5,686	5,700	5,683
Wt minigenes	586	586	586	586	586	586	586	586	586
Unique mutation comb.	4,938	4,886	4,865	4,923	4,923	4,927	4,929	4,942	4,926
**Model input**									
Comb. used by model	4,571	4,467	4,472	4,672	4,678	4,650	4,763	4,771	4,739
Excluded comb.	367 (7%)	419 (9%)	393 (8%)	251 (5%)	245 (5%)	277 (6%)	166 (3%)	171 (3%)	187 (4%)
Singlets	606	603	603	612	608	609	613	613	613
Doublets	1,009	1,000	1,001	1,023	1,025	1,021	1,034	1,032	1,030
Triplets	869	859	858	891	888	886	910	909	905
**Model output**									
Mutations in dataset	2,042	2,033	2,032	2,038	2,040	2,041	2,039	2,042	2,040
Estimated mutation effects	1,942 (95%)	1,915 (94%)	1,915 (94%)	1,957 (96%)	1,956 (96%)	1,957 (96%)	1,972 (97%)	1,974 (97%)	1,974 (97%)
Positions in dataset	680	679	680	680	680	680	680	680	680
Estimated position effects	676 (99.4%)	675 (99.6%)	676 (99.4%)	677 (99.6%)	677 (99.6%)	677 (99.6%)	677 (99.6%)	677 (99.6%)	677 (99.6%)

For each RNA-seq replicate (Rep), the internal library identifier is given together with information on the number of total and wild type (wt) minigene variants detected in each dataset, the number of unique mutation combinations (differentiated into those used or excluded from the model analysis; see Supplementary Note 2) as well as the used single-/double-/triple-mutation combinations (singlets/doublets/triplets, respectively). Output information summarises the mutation and position effects that can be estimated by the model in relation to all mutations and mutated positions represented in each dataset.

Supplementary Table 2. Summary of splicing-effective mutations and synergistic interactions with *HNRNPH* knockdown per region in HEK293T and MCF7 cells.

		Exon 10	Intron 10	Exon 11	Intron 11	Exon 12	Intron 12	Total
**HEK239T**	**Mutations**	555	261	441	240	498	42	2037
	Measured	487 (87.7%)	224 (85.8%)	381 (86.4%)	190 (79.2%)	430 (86.3%)	35 (83.3%)	1747 (85.8%)
	Any isoform > 5%	**117** (24%)	**118** (52.7%)	**270** (70.9%)	**108** (56.8%)	**144** (33.5%)	**21** (60%)	**778** (44.5%)
	AE inclusion	100	111	263	87	92	19	672
	AE skipping	20	67	185	53	29	9	363
	First IR	2	6	3	0	4	2	17
	Second IR	0	1	0	3	6	10	20
	Full IR	70	74	107	79	113	16	459
	Other	0	0	2	4	1	0	7
	Any isoform > 10%	**26** (5.3%)	**66** (29.5%)	**159** (41.7%)	**54** (28.4%)	**45** (10.5%)	**12** (34.3%)	**362** (20.7%)
	Any isoform > 20%	**2** (0.4%)	**32** (14.3%)	**59** (15.5%)	**25** (13.2%)	**9** (2.1%)	**9** (25.7%)	**136** (7.8%)
	**Positions**	185	87	147	80	166	14	679
	Measured	184 (99.5%)	87 (100%)	147 (100%)	77 (96.2%)	166 (100%)	14 (100%)	675 (99.4%)
	Any isoform > 5%	**92** (50%)	**67** (77%)	**134** (91.2%)	**64** (83.1%)	**99** (59.6%)	**13** (92.9%)	**469** (69.5%)
	Any isoform > 10%	**25** (13.6%)	**42** (48.3%)	**97** (66%)	**33** (42.9%)	**39** (23.5%)	**7** (50%)	**243** (36%)
	Any isoform > 20%	**2** (1.1%)	**18** (20.7%)	**45** (30.6%)	**16** (20.8%)	**9** (5.4%)	**4** (28.6%)	**94** (13.9%)

(a) Splicing-effective mutations (top) and positions (bottom) in HEK293T cells. The total number of possible and measured mutations/positions are indicated first, followed by the number of significant effects when considering any isoform at three cutoffs (>5%, >10% and >20%). Mutation effects are additionally given for each individual isoform. AE - alternative exon; IR - intron retention. Related to Fig. 2e and Supplementary Fig. 8.

Supplementary Table 2 (continued). Summary of splicing-effective mutations and synergistic interactions with *HNRNPH* knockdown per region in HEK293T and MCF7 cells.

		Exon 10	Intron 10	Exon 11	Intron 11	Exon 12	Intron 12	Total
MCF7	**Mutations**	555	261	441	240	498	42	2037
	Measured	501 (90.3%)	229 (87.7%)	386 (87.5%)	196 (81.7%)	440 (88.4%)	35 (83.3%)	1787 (87.7%)
	Any isoform >5%	**150** (29.9%)	**149** (65.1%)	**300** (77.7%)	**137** (69.9%)	**264** (60%)	**22** (62.9%)	**1022** (57.2%)
	AE inclusion	81	115	260	99	91	16	662
	AE skipping	86	125	271	102	217	18	819
	First IR	5	14	5	3	6	0	33
	Second IR	1	2	12	7	15	11	48
	Full IR	79	63	62	82	185	16	487
	Other	3	2	8	14	13	0	40
	Any isoform > 10%	**41** (8.2%)	**88** (38.4%)	**202** (52.3%)	**76** (38.8%)	**100** (22.7%)	**14** (40%)	**521** (29.2%)
	Any isoform > 20%	**6** (1.2%)	**39** (17%)	**86** (22.3%)	**32** (16.3%)	**16** (3.6%)	**10** (28.6%)	**189** (10.6%)
	**Positions**	185	87	147	80	166	14	679
	Measured	185 (100%)	87 (100%)	147 (100%)	78 (97.5%)	166 (100%)	14 (100%)	677 (99.7%)
	Any isoform > 5%	**108** (58.4%)	**74** (85.1%)	**139** (94.6%)	**70** (89.7%)	**147** (88.6%)	**12** (85.7%)	**550** (81.2%)
	Any isoform > 10%	**36** (19.5%)	**52** (59.8%)	**112** (76.2%)	**48** (61.5%)	**74** (44.6%)	**8** (57.1%)	**330** (48.7%)
	Any isoform > 20%	**6** (3.2%)	**22** (25.3%)	**61** (41.5%)	**22** (28.2%)	**14** (8.4%)	**5** (35.7%)	**130** (19.2%)

(b) Splicing effective mutations (top) and positions (bottom) in MCF7 cells. Format as in (a).

Supplementary Table 2 (continued). Summary of splicing-effective mutations and synergistic interactions with *HNRNPH* knockdown per region in HEK293T and MCF7 cells.

		Exon 10	Intron 10	Exon 11	Intron 11	Exon 12	Intron 12	Total
**MCF7 – synergistic interactions with *HNRNPH* knockdown**	**Mutations**	555	261	441	240	498	42	2037
	Measured	501 (90.3%)	229 (87.7%)	385 (87.3%)	196 (81.7%)	440 (88.4%)	35 (83.3%)	1786 (87.7%)
	Any isoform |*z*| > 2	**70** (14%)	**35** (15.3%)	**135** (35.1%)	**51** (26%)	**58** (13.2%)	**5** (14.3%)	**354** (19.8%)
	AE skipping	37	21	100	17	39	1	215
	First IR	8	3	5	4	8	1	29
	Second IR	10	6	6	4	7	0	33
	Full IR	30	20	47	14	18	3	132
	Other	21	17	54	30	9	1	132
	Any isoform |*z*| > 3	**44** (8.8%)	**25** (10.9%)	**89** (23.1%)	**31** (15.8%)	**31** (7%)	**2** (5.7%)	**222** (12.4%)
	Any isoform |*z*| > 5	**10** (2%)	**3** (1.3%)	**35** (9.1%)	**7** (3.6%)	**11** (2.5%)	**0** (0%)	**66** (3.7%)
	**Positions**	185	87	147	80	166	14	679
	Measured	185 (100%)	87 (100%)	147 (100%)	78 (97.5%)	166 (100%)	14 (100%)	677 (99.7%)
	Any isoform |*z*| > 2	**61** (33%)	**28** (32.2%)	**93** (63.3%)	**38** (48.7%)	**54** (32.5%)	**4** (28.6%)	**278** (41.1%)
	Any isoform |*z*| > 3	**42** (22.7%)	**23** (26.4%)	**61** (41.5%)	**25** (32.1%)	**31** (18.7%)	**2** (14.3%)	**184** (27.2%)
	Any isoform |*z*| > 5	**10** (5.4%)	**3** (3.4%)	**27** (18.4%)	**7** (9%)	**11** (6.6%)	**0** (0%)	**58** (8.6%)

(c) Synergistic interactions between mutations (top) or positions (bottom) and *HNRNPH* knockdown in MCF7 cells. Same format as in (a). Interactions for any isoform are reported at different absolute *z*-score cutoffs ($|z|$ >2, >3 and >5). Note that synergistic interactions are calculated from ratios of a given isoform over AE inclusion, so no synergistic interactions are given for AE inclusion. Related to Fig. 5c and Supplementary Fig. 12c.

Supplementary Table 3: Association of *HNRNPH2* expression with *RON* exon 11 inclusion levels in different TCGA cohorts. Related to Fig. 3f.

TCGA cohort	# samples	Spearman correlation	*HNRNPH2* variance	*P*-value	FDR	Significance (FDR < 0.05)
BRCA	778	-0.28	0.24	1.5e-15	3.9e-14	TRUE
LUAD	485	-0.25	0.15	3.5e-08	4.6e-07	TRUE
COAD	323	-0.27	0.16	8.0e-07	6.9e-06	TRUE
READ	103	-0.41	0.16	2.0e-05	1.3e-04	TRUE
ESCA	181	-0.29	0.13	7.3e-05	3.8e-04	TRUE
PAAD	163	-0.29	0.08	2.2e-04	9.5e-04	TRUE
LUSC	315	-0.2	0.12	3.8e-04	1.4e-03	TRUE
CESC	248	-0.2	0.16	1.3e-03	4.2e-03	TRUE
STAD	414	-0.14	0.13	3.2e-03	9.2e-03	TRUE
HNSC	455	-0.13	0.16	4.3e-03	1.1e-02	TRUE
THYM	51	-0.37	0.10	7.0e-03	1.7e-02	TRUE
OV	178	-0.18	0.14	1.5e-02	3.3e-02	TRUE
KIRC	11	-0.56	0.25	7.0e-02	1.4e-01	FALSE
PRAD	10	-0.52	0.03	1.3e-01	2.4e-01	FALSE
TGCT	17	0.36	0.10	1.5e-01	2.6e-01	FALSE
BLCA	251	-0.086	0.17	1.7e-01	2.8e-01	FALSE
THCA	282	-0.077	0.04	1.9e-01	2.9e-01	FALSE
KIRP	47	-0.19	0.10	2.1e-01	3.0e-01	FALSE
CHOL	28	-0.2	0.09	3.1e-01	4.2e-01	FALSE
LIHC	24	-0.21	0.15	3.2e-01	4.2e-01	FALSE
SKCM	59	-0.11	0.14	4.0e-01	5.0e-01	FALSE
KICH	4	-0.6	0.18	4.2e-01	5.0e-01	FALSE
UCEC	61	-0.09	0.19	4.9e-01	5.5e-01	FALSE
SARC	19	0.076	0.37	7.6e-01	8.2e-01	FALSE
DLBC	3	0.5	0.04	1	1	FALSE
LAML	3	0.5	0.08	1	1	FALSE
GBM	1	NA	NA	NA	NA	NA

Cancer types: BLCA, Bladder Urothelial Carcinoma; BRCA, Breast Invasive Carcinoma; CESC, Cervical Squamous Cell Carcinoma and Endocervical Adenocarcinoma; CHOL, Cholangiocarcinoma; COAD, Colon Adenocarcinoma; DLBC, Lymphoid Neoplasm Diffuse Large B-cell Lymphoma; ESCA, Esophageal Carcinoma; GBM, Glioblastoma Multiforme; HNSC, Head-Neck Squamous Cell Carcinoma; KICH, Kidney Chromophobe; KIRC, Kidney Renal Clear Cell Carcinoma; KIRP, Kidney Renal Papillary Cell Carcinoma; LAML, Acute Myeloid Leukemia; LIHC, Liver Hepatocellular Carcinoma; LUAD, Lung Adenocarcinoma; LUSC, Lung Squamous Cell Carcinoma; OV, Ovarian Serous Cystadenocarcinoma; PAAD, Pancreatic Adenocarcinoma; PRAD, Prostate Adenocarcinoma; READ, Rectum Adenocarcinoma; SARC, Sarcoma; SKCM, Skin Cutaneous Melanoma; STAD, Stomach Adenocarcinoma; TGCT, Testicular Germ Cell Tumours; THCA, Thyroid Carcinoma; THYM, Thymoma; UCEC, Uterine Corpus Endometrial Carcinoma.

Supplementary Table 4: Oligonucleotides used in this study.

Name	Sequence (5’-3’)	Purpose
minigene_cloning_fwd	CCCAAGCTTTGTGAGAGGCAGCTTCCAGA	Cloning of wt *RON* minigene
minigene_cloning_rev	CAGTCTAGANNNNNNNNNNNNNNNGGATCCGCC ATTGGTTGGGGGTAGG-GGCTGATTAAAGGTAGG	Cloning of wt *RON* minigene
BamHI_HNRNPH1_fwd	catGGATCCaccatgatgttgggcacggaagg	Cloning of HNRNPH1 overexpression construct
XbaI_HNRNPH1_rev	cattctagactatgcaatgtttgattgaaaatc	Cloning of HNRNPH1 overexpression construct
RT-PCR_minigene_fwd	TGCCAACCTAGTTCCACTGA	RT-PCR for *RON* minigene
RT-PCR_minigene_rev	GCAACTAGAAGGCACAGTCG	RT-PCR for *RON* minigene
RT-PCR_endo_fwd	CCTGAATATGTGGTCCGAGACCCCCAG	RT-PCR for endogenous *RON* gene
RT-PCR_endo_rev	CTAGCTGCTTCCTCCGCCACCAGTA	RT-PCR for endogenous *RON* gene
RON A	CAAGCAGAAGACGGCATACGAGATCGGTCTCGGC ATTCCTGCTGAACCGCTCTTCCGATCTNNNNNNNN NNCTATAGGGAGACCCAAGCTT	Illumina fwd sequencing primer for DNA-seq
RON B	CAAGCAGAAGACGGCATACGAGATCGGTCTCGGC ATTCCTGCTGAACCGCTCTTCCGATCTNNNNNNNN NNGTTCCACTGAAGCCTGAG	Illumina fwd sequencing primer for DNA-seq and RNA-seq
RON C	CAAGCAGAAGACGGCATACGAGATCGGTCTCGGC ATTCCTGCTGAACCGCTCTTCCGATCTNNNNNNNN NNAGCTGCCAGCACGAGTTC	Illumina fwd sequencing primer for DNA-seq
RON D	CAAGCAGAAGACGGCATACGAGATCGGTCTCGGC ATTCCTGCTGAACCGCTCTTCCGATCTNNNNNNNN NNGAATCTGAGTGCCCGAGG	Illumina fwd sequencing primer for DNA-seq
RON E	CAAGCAGAAGACGGCATACGAGATCGGTCTCGGC ATTCCTGCTGAACCGCTCTTCCGATCTNNNNNNNN NNctactggctggtcctcatga	Illumina fwd sequencing primer for DNA-seq
P5 SOLEXA RON	AATGATACGGCGACCACCGAGATCTACACTCTTTCC CTACACGACGCTCTTCCGATCTNNNNNNNNNNAT AGAATAGGGCCCTCTAGA	Illumina rev sequencing primer for DNA-seq and RNA-seq
RT1	NNAATANNNAGATCGGAAGAGCGTCGTGGATCCT GAACCGC	RT primer HNRNPH iCLIP for wt replicate 1
RT2	NNTTTCNNNAGATCGGAAGAGCGTCGTGGATCCT GAACCGC	RT primer HNRNPH iCLIP for wt replicate 2
RT3	NNCGATNNNAGATCGGAAGAGCGTCGTGGATCCT GAACCGC	RT primer HNRNPH iCLIP for wt replicate 3
RT4	NNTTCTNNNAGATCGGAAGAGCGTCGTGGATCCT GAACCGC	RT primer HNRNPH iCLIP for G305A replicate 1
RT5	NNCTCGNNNAGATCGGAAGAGCGTCGTGGATCCT	RT primer HNRNPH iCLIP

	GAACCGC	for G305A replicate 2
RT6	NNACGCNNNAGATCGGAAGAGCGTCGTGGATCCT GAACCGC	RT primer HNRNPH iCLIP for G305A replicate 3
RT7	NNTTCTNNNAGATCGGAAGAGCGTCGTGGATCCT GAACCGC	RT primer HNRNPH iCLIP for G331C replicate 1
RT8	NNGGCGNNNAGATCGGAAGAGCGTCGTGGATCCT GAACCGC	RT primer HNRNPH iCLIP for G331C replicate 2
RT9	NNTGTGNNNAGATCGGAAGAGCGTCGTGGATCCT GAACCGC	RT primer HNRNPH iCLIP for G348C replicate 1
RT10	NNGTATNNNAGATCGGAAGAGCGTCGTGGATCCT GAACCGC	RT primer HNRNPH iCLIP for G348C replicate 2

Oligonucleotides were purchased either from Sigma-Aldrich or Integrated DNA Technologies.

Supplementary Figures 1 - 16

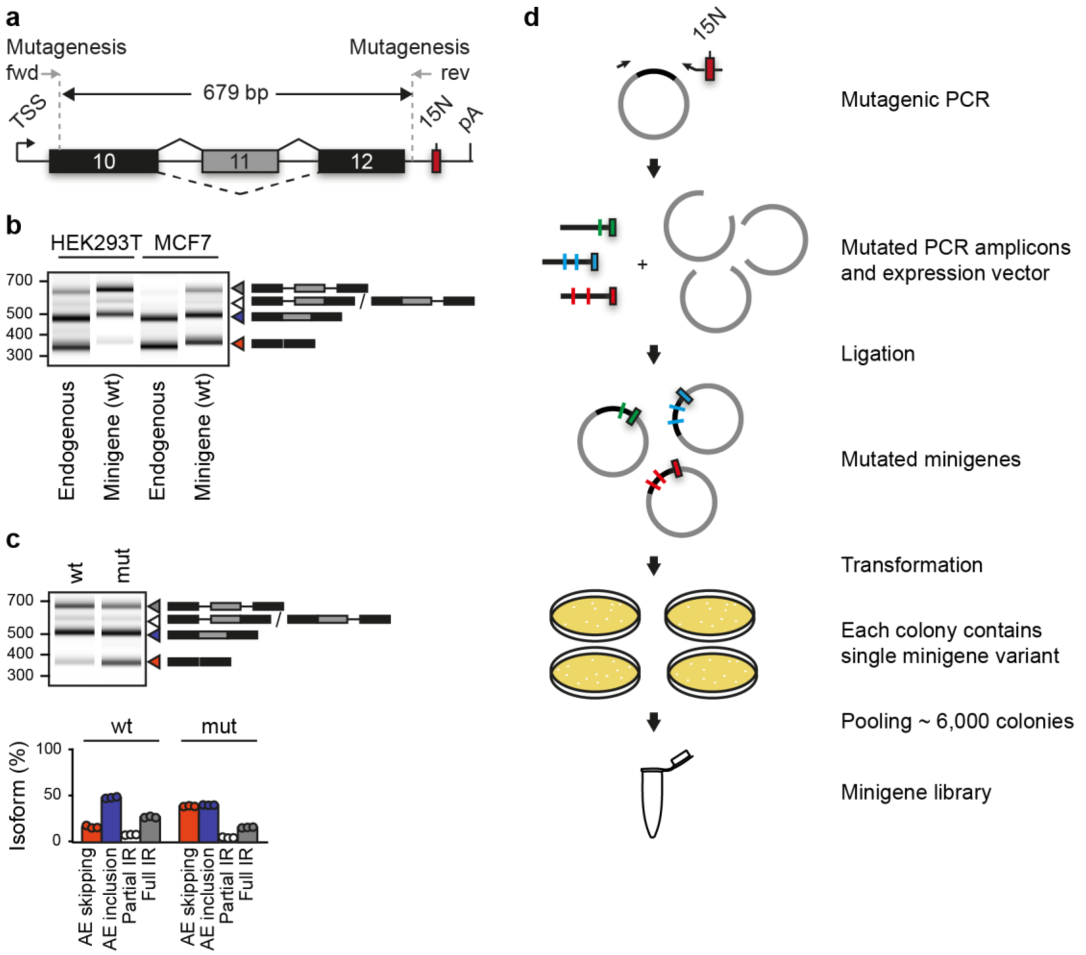

Supplementary Figure 1: Random mutagenesis generates a mutated *RON* minigene library. Related to Fig. 1a.

(a) The *RON* minigene harbours genomic sequence of the *RON* gene (*MST1R*, ENSG00000164078) including alternative exon 11 with the complete flanking introns and constitutive exons 10 and 12 (chr3: 49,933,098 - 49,933,837, GRCh37/hg19). Mutagenesis of a 679 bp region was performed using error-prone PCR and indicated forward (fwd) and reverse (rev) primers. TSS, transcriptional start site; pA, polyadenylation site; 15N, the 15-nt barcode as a unique identifier of each minigene variant.

(b) The wild type (wt) *RON* minigene gives rise to the same splicing isoforms as the endogenous *RON* gene in HEK293T and MCF7 cells. Gel-like representation of capillary electrophoresis of PCR products from semiquantitative RT-PCR monitoring *RON* exon 11 inclusion. Note that different primer combinations were used to differentiate between the endogenous *RON* gene and the *RON* wt minigene (**Supplementary Table 4**), resulting in a 52-bp difference in the RT-PCR products for the same isoforms.

(c) Introducing a previously published triple mutation[3] into the *RON* minigene (T565A, G566T, G569A; mut) triggers the expected splicing response. Gel-like representation of RT-PCR products from HEK293T cells as in (b). Bar diagram below shows quantification of isoform frequencies (in %) for alternative exon (AE) inclusion and skipping, as well as partial and full intron retention (IR). Individual data points from three independent biological replicates are displayed. Note that partial IR refers to the sum of first IR and second IR isoforms that cannot be discriminated in the RT-PCR analysis.

(d) Schematic overview of the experimental procedure to generate the mutated minigene library. Mutagenic PCR amplification of the wt *RON* minigene creates mutated amplicons that were ligated into the expression vector to obtain the mutated minigene library. The reverse primer used in the mutagenic PCR carries a 15-nt random sequence (15N) that is included as a unique identifier into each minigene variant. See Methods for details. Coloured vertical bars schematically indicate point mutations.

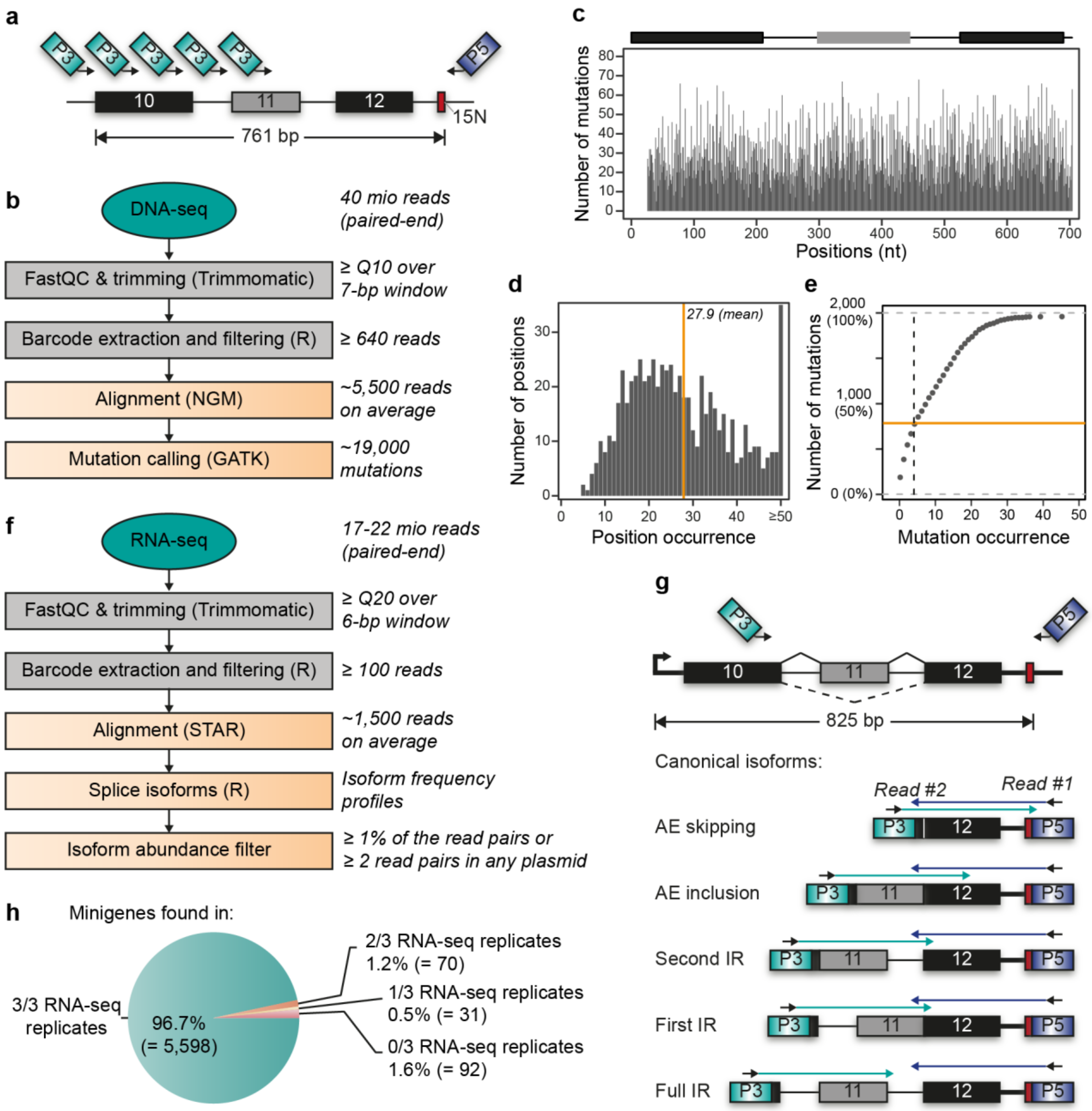

Supplementary Figure 2: Mutations and splicing products from the minigene library are characterised by high-throughput DNA and RNA sequencing. Related to Fig. 1.

(a) Schematic of amplicons for paired-end DNA sequencing. Reverse primer binds downstream of 15-nt barcode (15N, red box) and introduces Illumina sequencing adaptor P5 (Read #1). Five variants of the forward primer bind to subsequent positions resulting in five overlapping amplicons of the minigene. Forward primers introduce P3 (Read #2).

(b) Bioinformatics workflow for DNA-seq analysis to characterise mutations. Quality control and trimming was performed with FastQC and Trimmomatic, respectively, followed by custom scripts (in R) to extract 15-nt barcode and filter for minigenes with ≥640 read pairs. Reads were aligned to wt *RON* minigene sequence using NextGenMap (NGM), and mutation calling was done using HaplotypeCaller tool from Genome Analysis Toolkit (GATK). See Methods for details.

(c) 18,948 point mutations evenly distribute across the *RON* minigene. Bar diagram showing the number of minigene variants (out of 5,791) harbouring a mutation in a given position.

(d) Each position is on average mutated in 28 different minigene variants. Histogram summaries number of positions with a given mutation frequency. Orange line indicates mean mutation frequency across all positions.

(e) The majority of mutations occur in at least five different plasmid variants (labelled in orange). Cumulative distribution of mutations with a given mutation occurrence.

(f) Bioinformatics workflow for RNA-seq analysis to quantify splice isoforms. Upon quality control and filtering similar to (b), reads were aligned to wt *RON* minigene using splice-aware alignment software STAR. All isoforms present in RNA-seq library were reconstructed and filtered for minimum abundance using custom scripts (R). See Methods for details.

(g) Each canonical isoform is uniquely identified by paired-end RNA-seq. Read #1 starting from the P5 adaptor provides the 15-nt barcode information and the splice junction upstream of exon 12, while Read #2 from P3 reads the splice junction downstream of exon 10. For partial or full IR isoforms, both reads extend into the respective intron.

(h) The majority of minigene variants in the library is recovered in all three RNA-seq replicates from HEK293T cells. Pie chart displays the fraction of 5,791 minigene variants in the library that is recovered in 0-3 replicates.

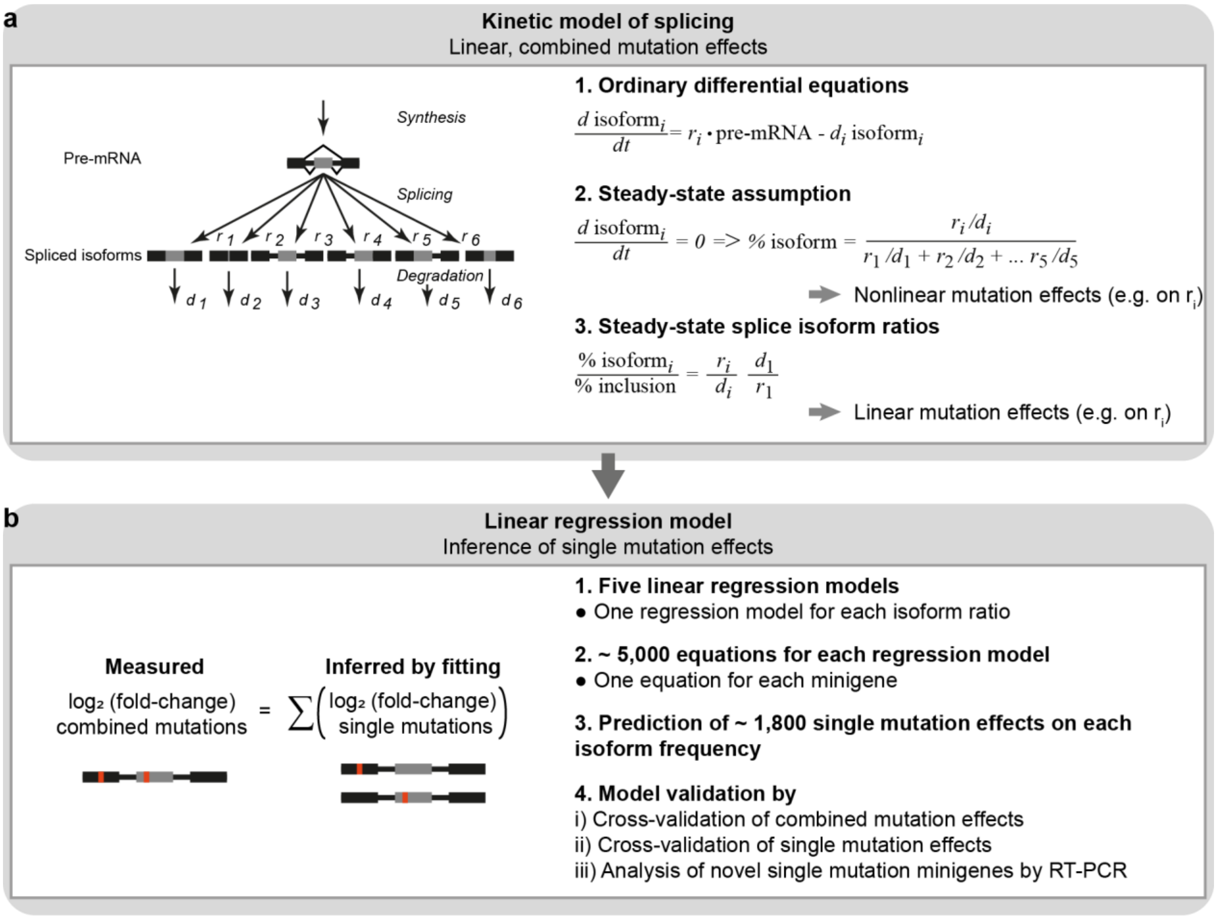

Supplementary Figure 3: Modelling workflow for the inference of single mutation effects. Related to Fig. 2a.

(a) Kinetic model of splicing linearises splicing effects. Pre-mRNA synthesis, splicing reactions and mRNA degradation (scheme) are described by a set of ordinary differential equations (1). At steady state, each isoform frequency is described by a Michaelis-Menten-type equation (2), leading to non-linear mutation effects (e.g., effect of a mutation-induced change in r_1 depends on other parameters, i.e. other mutations). Mutation effects (e.g., on r_1) have linear effects when splice isoform ratios relative to a reference isoform are considered (3). See **Supplementary Note 1** for details.

(b) Linear regression model infers single mutation effects. Effect of combined mutations (log fold-change) is formulated as sum of individual mutation effects. Five regression models (one per splice isoform), each containing ~5,000 equations (one per minigene), are formulated and fitted to the data. The models can be used to predict ~2,000 single mutation effects (700 nucleotides * 3 nucleotide exchanges) for each splice isoform. Model was subjected to cross-validation by leaving out 10% of the minigenes (i) or individual single mutation minigenes (ii) from the fit. Independent validation was performed by testing model predictions against RT-PCR for novel single mutation minigenes. See **Supplementary Note 2** for details.

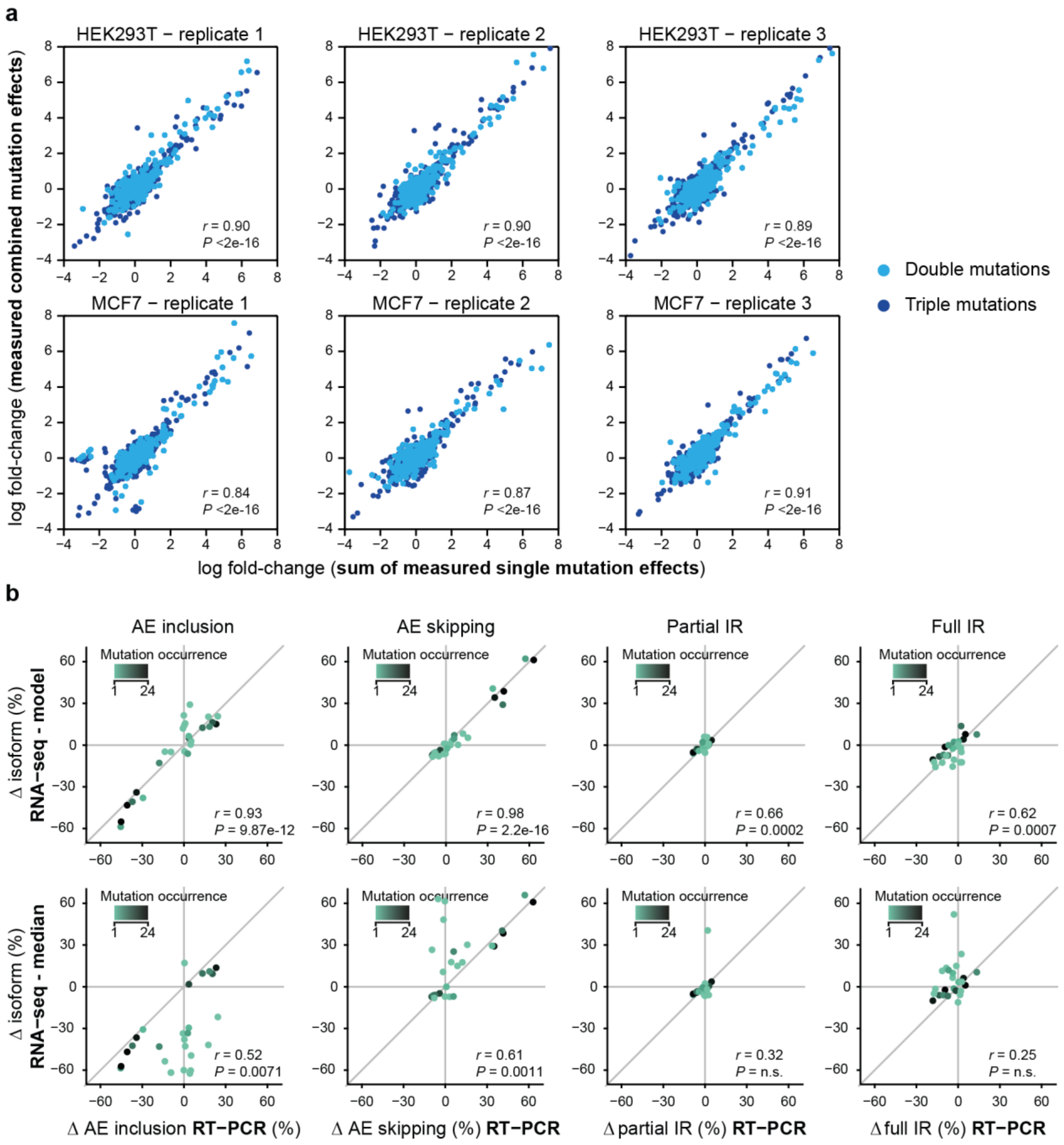

Supplementary Figure 4: Single mutation effects are additive and confirmed by semiquantitative RT-PCR. Related to Fig. 2d.

(a) Single mutation effects are additive. Scatterplots show that sum of directly measured single mutation effects (from single-mutation minigenes; according to linear regression assumption, **Fig. 2a**; x-axes) agrees well with corresponding experimental measurements (y-axes) of minigenes containing two or three of these mutations (double and triple mutations, respectively). Analyses are shown for three replicates in HEK293T (top) and MCF7 cells (bottom).

(b) Regression model outperforms a median-based estimation of single mutation effects. Effects of mutations that rarely occur in the library (colour-coded) correlate better with model-inferred than median-based estimates. Scatterplots compare model-inferred (top row) and median-based (bottom row) estimations of single mutation effects relative to wt (y-axes) to semiquantitative RT-PCR measurements (x-axes) of targeted minigenes harbouring single point mutations, insertions and deletions (**Supplementary Data 8**). Separate plots are shown for different splice isoforms. First IR and second IR were summed up as ‘partial IR’, since these isoforms cannot be discriminated in RT-PCR. Pearson correlation coefficient and associated *P*-value are given in each panel. See Methods for description of median-based estimation.

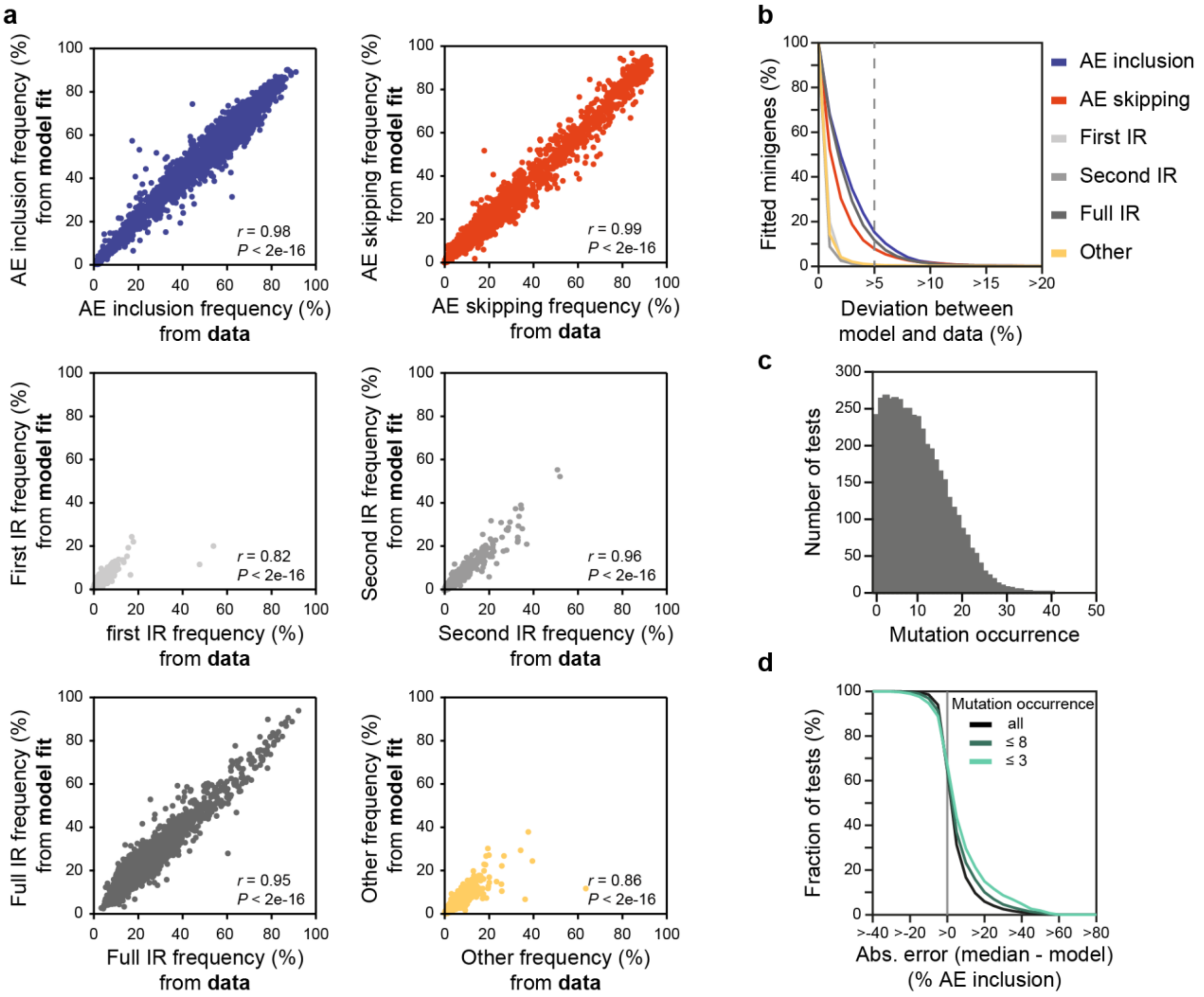

Supplementary Figure 5: The regression model increases the precision of isoform frequency estimations. Related to Fig. 2.

(a) Regression model describes experimentally measured isoform frequencies for each mutated minigene variant with high correlation (Pearson correlation coefficients *r* = 0.82-0.99, *P*-values < 2e-16). Scatterplots show frequencies of each of five canonical and non-canonical ('other') isoforms for combined mutations calculated from fitted model against measured data of one biological replicate (see **Supplementary Note 2**). Related to Fig. 2b.

(b) Majority of minigene variants are fitted within 5% deviation from measured value. For each isoform, fraction of fitted minigenes (y-axis) is shown for which model-derived isoform frequencies and measured data deviate more than a given %-value (x-axis). Related to Fig. 2b.

(c) Number of tests for different mutation occurrences that was used to calculate inference error of the model shown in Fig. 2c. Inference errors were estimated by separately benchmarking 561 mutation effects from single-mutation minigenes. To this end, minigenes containing the respective mutation were successively removed from the dataset, and subsequently model-inferred mutation effects were compared to isoform frequencies of single-mutation minigenes excluded from the analysis. Mutation occurrence shows number of different multi-mutation minigenes containing reference mutation used in one test. By successively reducing the dataset, we obtain the prediction accuracy for a particular mutation for different mutation occurrences. In some cases, estimation of mutational effects was not possible from a reduced dataset. These tests were left out, which explains the non-monotonical dependence of the number of tests on mutation occurrence. Related to Fig. 2c.

(d) Gain in accuracy for model-inferred isoform frequency estimations compared to median-based estimates. Difference of absolute errors in AE inclusion (%) between model and median-based calculation (x-axis) for a cumulative fraction of tests (y-axis) used in Fig. 2c. In 65% of tests, the model outperforms median-based estimation. Improvement of the model is more pronounced when considering only tests with low mutation occurrences (see legend). Related to Fig. 2c.

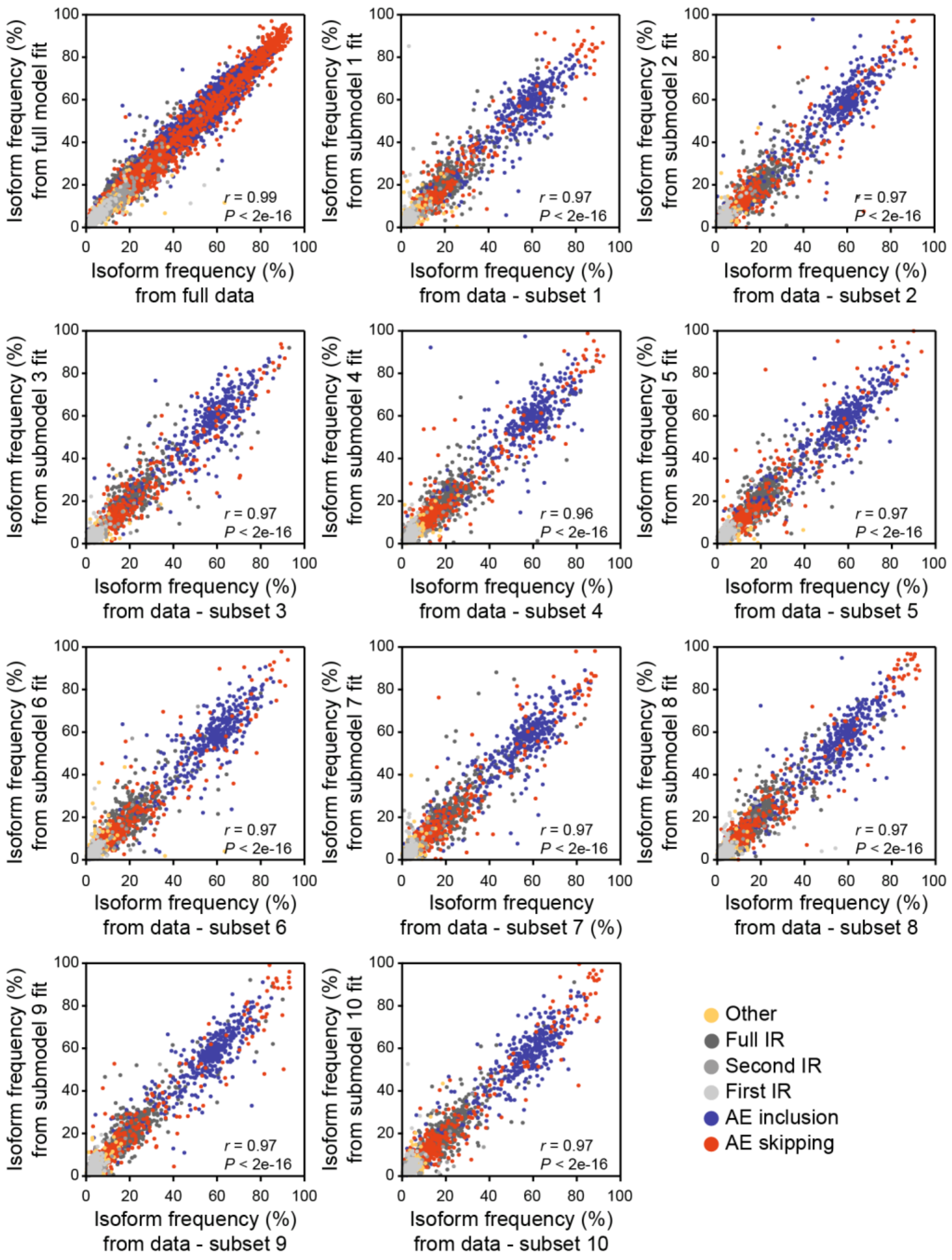

Supplementary Figure 6: Cross-validation underlines predictive power of the model for minigenes that were not used in training. Related to Fig. 2b.

The minigene library was randomly split into ten equal-sized subsets. During 10-fold cross-validation, regression models (one for each splice isoform) were fitted to all data excluding one subset. Scatterplots compare model-predicted splicing outcome for left-out subset to corresponding experimental data for all splice isoforms (see legend). In the first panel, full model fit is plotted against full dataset, followed by model prediction-data comparisons for ten different subsets. Pearson correlation coefficient and associated *P*-value are given in each panel.

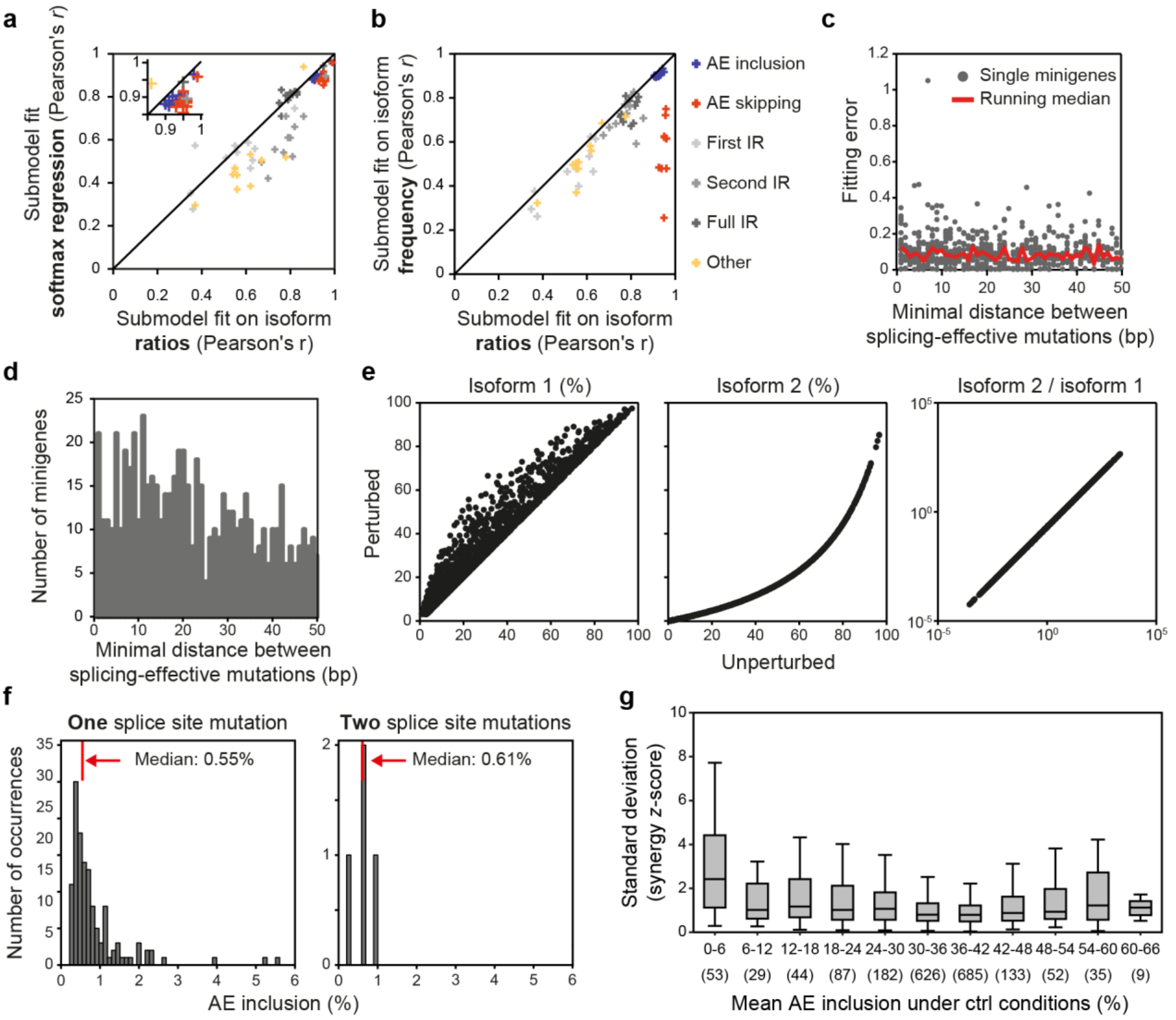

Supplementary Figure 7: Linear regression modelling based on splice isoform ratios accurately infers single mutation effects in HEK293T cells. Related to Fig. 5b.

(a,b) Correlation between model-inferred isoform frequencies and experimental data improves when using linear regression on isoform ratios compared to softmax regression **(a)** or constrained linear regression on isoform frequencies **(b)**. Comparison of Pearson correlation coefficients (*r*) from 10-fold cross-validation (see **Supplementary Note 2**). Isoforms are colour-coded as indicated in (b).

(c) Distance between mutations in the *RON* minigene does not influence the fitting error. Overall fitting error was computed by summing up the absolute deviation between fit and data for all isoforms. Only minigenes containing at least two mutations with significant effects on either isoform are plotted. The minimal distance between adjacent effective mutations contained in each minigene defines the x-axis.

(d) While 1,682 minigenes in our screen contained at least two splicing-effective mutations, only 84 of them occur within a distance of less than seven nucleotides. Histogram quantifies minigenes with a given minimal distance of splicing-effective mutations, corresponding to the number of data points for each value on the x-axis plotted in (c).

(e) Numeric simulation of competing splicing kinetic reactions reveals that perturbations of splicing rates have a linear effect on splice isoform ratios. Kinetic equations reflecting competing splicing reactions (Supplementary Equations 1 and 2 in **Supplementary Note 1**) were analysed *in silico*. The change of the steady-state after decreasing the production rate of one splicing isoform to 20% was simulated. The effect of this perturbation on all splicing isoforms is nonlinear and depends on the mutational context. In contrast, the perturbation has a linear effect on splice isoform ratios.

(f) The presence of two splice site mutations in a minigene does not further decrease AE inclusion compared to minigenes containing only one splice site mutation. Histograms of AE inclusion frequency in minigenes containing one or two splice site mutations.

(g) Computation of the synergy score is unstable for mutations abolishing AE inclusion. Boxplox shows the standard deviation of the synergy score for the AE skipping to AE inclusion ratio over the three replicates for mutations with mean control AE inclusion in different ranges. Bounds of each box represent quartiles, centre line denotes 50th percentile, and whiskers extend to most extreme data points. Mutations leading to control AE inclusion less than 6% show greatest uncertainty in the computation of the synergy *z*-score.

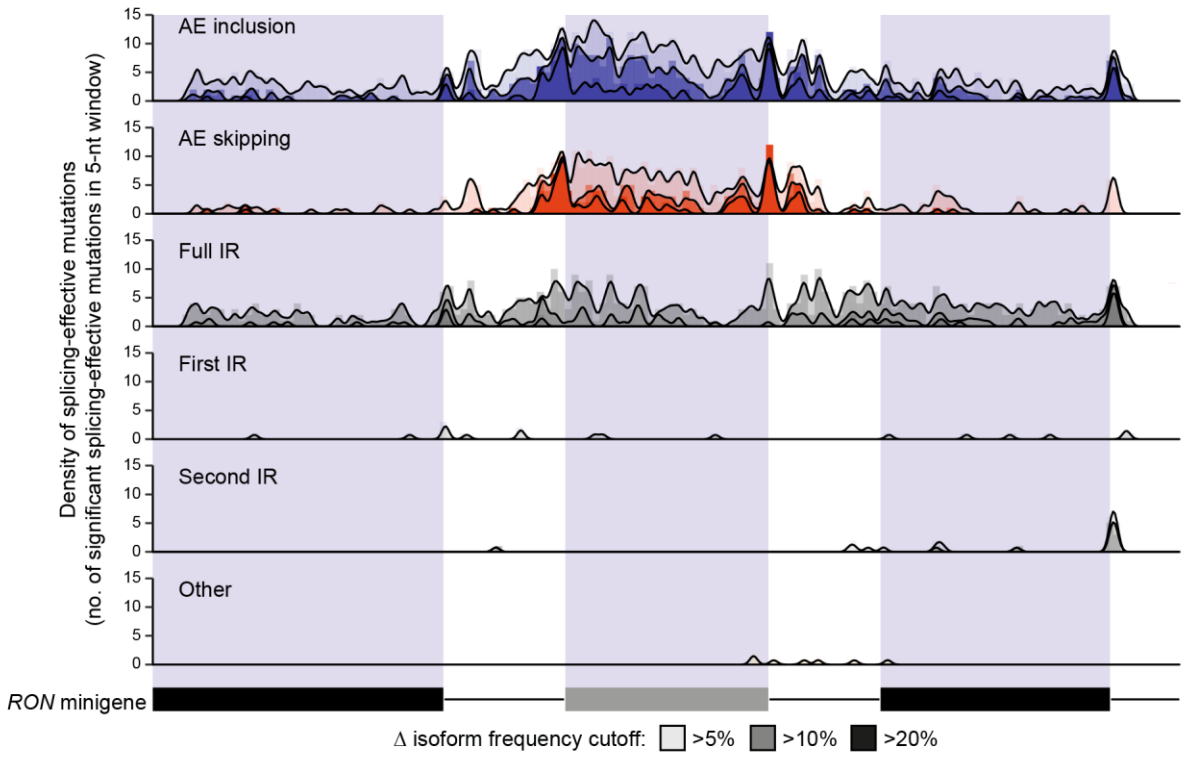

Supplementary Figure 8: Complete landscape of splicing-effective mutations in HEK293T cells. Related to Fig. 2e.

Bar diagrams for each isoform show the number of splicing-effective mutations in adjacent 5-nt windows across the *RON* minigene (FDR < 0.1%). Lines indicate the density of significant splicing-effective mutations in a 5-nt sliding window. Light to dark shading indicates cutoffs at >5%, >10%, and >20% change in isoform frequency, identifying a total of 778, 362 and 136 splicing-effective mutations, respectively. The alternative exon constitutes a regulatory hotspot for alternative exon (AE) inclusion and AE skipping. Mutations affecting full intron retention (IR) are dispersed across the alternative exon and the downstream constitutive exon.

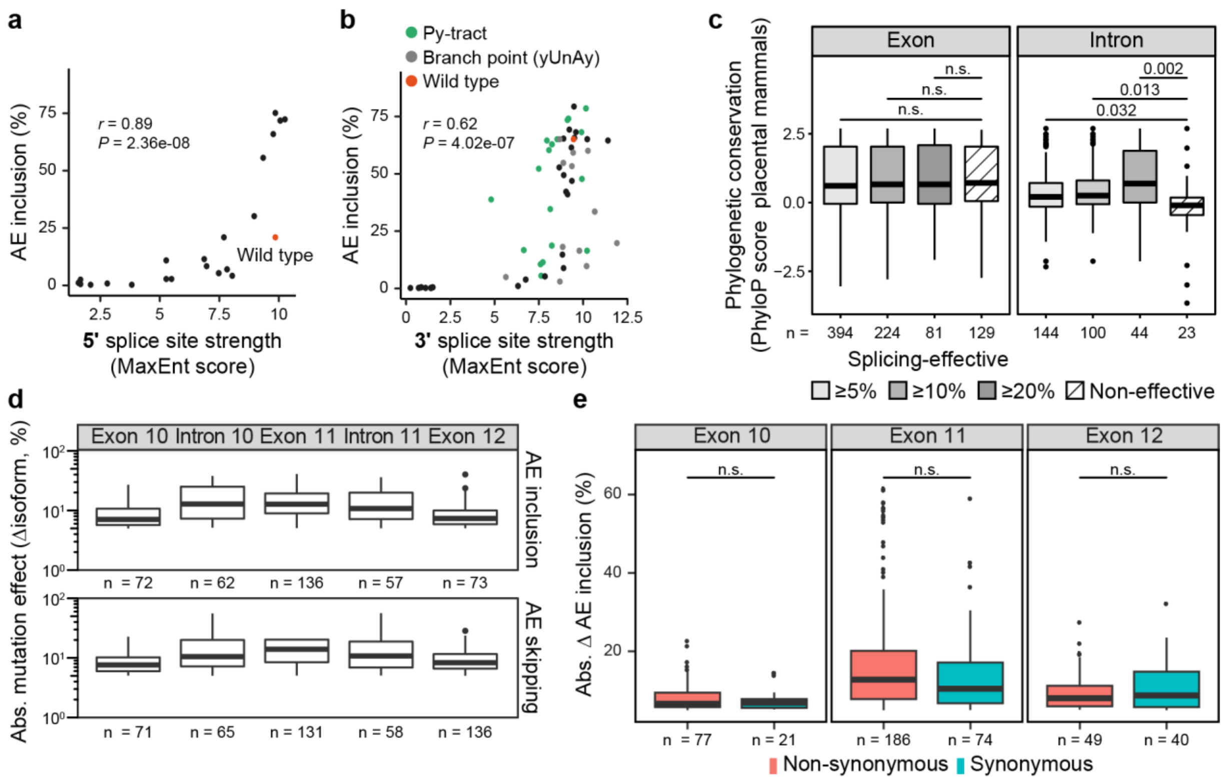

f

	Effective mutations	Non-effective mutations	Percentage of effective mutations
Non-synonymous	312	568	35%
Synonymous	135	272	33%

Supplementary Figure 9: Splice site strength, evolutionary conservation and coding potential of splicing-effective positions. Related to Fig. 2.

(a,b) Mutation effects at the 3' splice site **(a)** and 5' splice site **(b)** of *RON* exon 11 correlate with splice site strengths predicted by the MaxEntScan software. Scatterplots compare AE inclusion frequencies from HEK293T cells (y-axes) to the predicted splice site strength (MaxEnt score) for all mutations in positions considered by MaxEntScan (278-300 nt and 442-450 nt for 3' and 5' splice site, respectively). Red, green and grey dots indicate wt minigene and variants with mutations in polypyrimidine tract (Py-tract; 286-293 nt) and branch point motif (yUnAy, where y is pyrimidine and n is any base; 279-283 nt), respectively. *r*, Spearman correlation coefficient and corresponding *P*-value.

(c) Splicing-effective positions are significantly more conserved evolutionarily than permissive mutations within introns, but not exons. Boxplot shows distribution of conservation scores (PhyloP score across 46 placental mammals) for splicing-effective (light to dark shading indicating cutoffs at >5%, >10%, and >20% change in isoform frequency) and permissive positions in MCF7 cells in exons (left) and introns (right) of the *RON* minigene. Number of positions in each box indicated below. Centre line and bounds of each box denote 25th, 50th and 75th percentile, and whiskers extend to most extreme values within 1.5x interquartile range (IQR). *P*-values correspond to two-sided Mann-Whitney-Wilcoxon test. n.s., not significant.

(d) Splicing-effective mutations in *RON* exon 11 and the flanking introns are comparably strong. Boxplots summarise absolute changes in AE inclusion (top) or AE skipping (bottom) for significant splicing-effective mutations (>5%) in the different transcript regions (number given below). Box representation as in (c).

(e) Synonymous and non-synonymous mutations show similar effect sizes. Boxplots show absolute changes in AE inclusion in HEK293T cells for synonymous and non-synonymous mutations in exons 10-12. Number of positions in each box indicated below. Box representation as in (c). Significance was tested using two-sided Mann-Whitney-Wilcoxon test. n.s., not significant.

(f) Significant splicing-regulatory effects are observed with equal frequency among synonymous and non-synonymous mutations. Table summarises coincidence of significant splicing effects in HEK293T cells and synonymous/non-synonymous mutations across the three exons of the *RON* minigene.

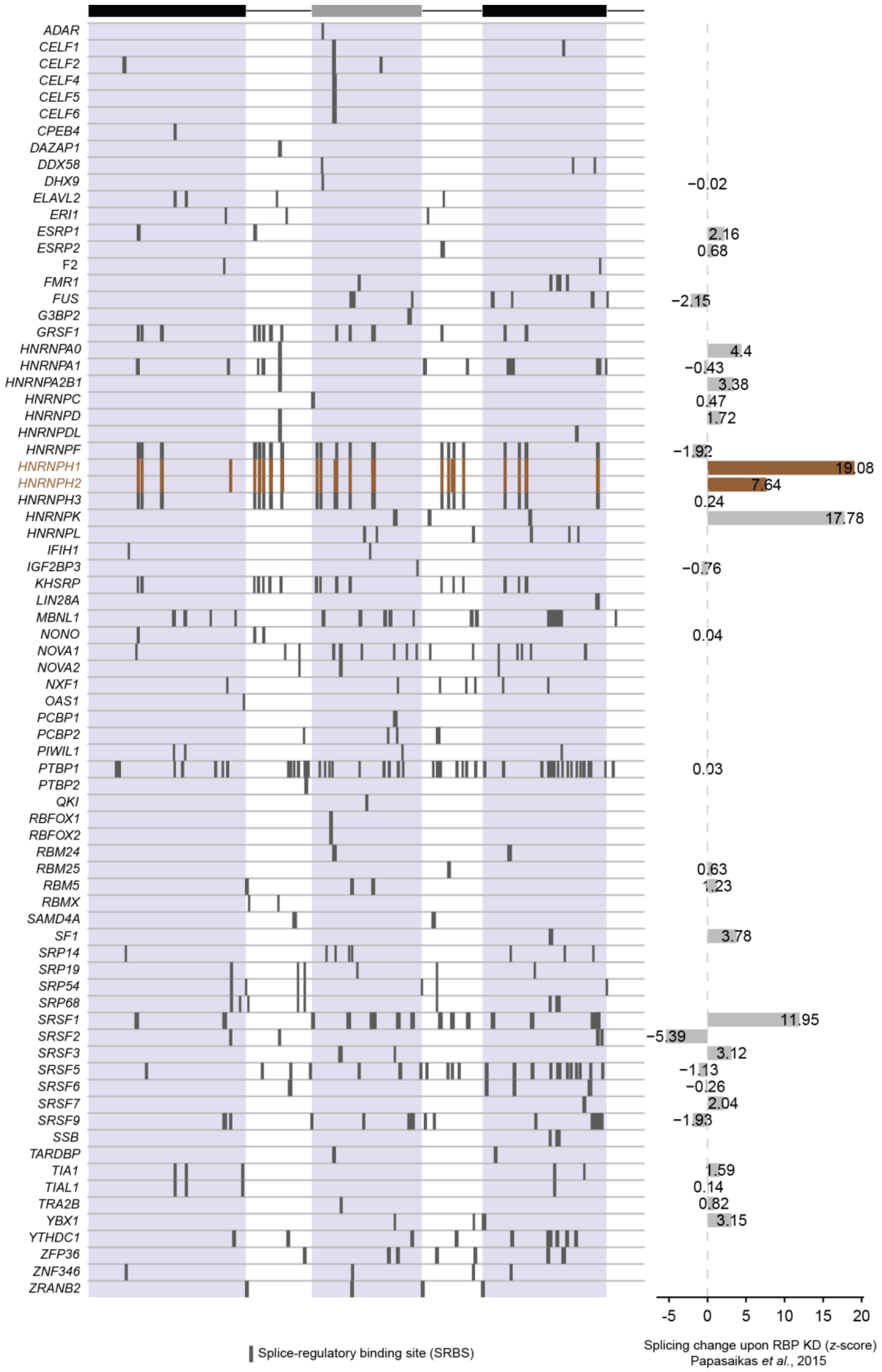

Supplementary Figure 10: Putative RBP regulators of *RON* exon 11 splicing and their predicted splice-regulatory binding sites. Related to Fig. 3e.

in silico binding site predictions for RNA-binding proteins (RBPs) identify splice-regulatory binding sites (SRBS; predicted binding sites that show substantial mutation effects, see Methods). Boxes indicate the location of SRBS for the 76 putative RBP regulators that were identified by ATtRACT. Predicted binding sites for HNRNPH1 and HNRNPH2 are highlighted in brown. Bar diagram (right) shows splicing effects (*z*-scores, values indicated at each bar) for 31 RBPs that are present in published data[2] on *RON* exon 11 splicing upon RBP knockdown (KD). Positive and negative *z*-scores correspond to increased and decreased *RON* exon 11 inclusion upon RBP KD, respectively.

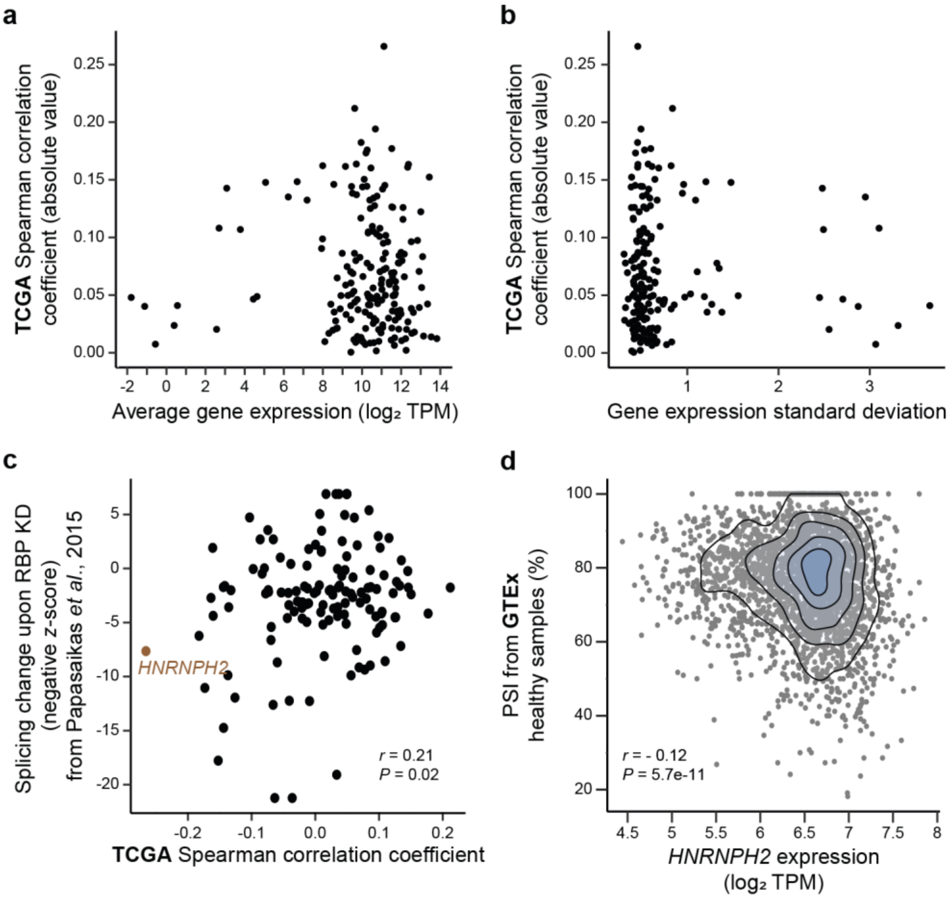

Supplementary Figure 11: Expression correlation of *HNRNPH2* and other RNA-binding proteins (RBPs) with *RON* exon 11 inclusion in TCGA and GTEx samples. Related to Fig. 3c,d,f.

(a,b) Absolute Spearman correlation coefficients of RBP expression (in transcripts per million, TPM) and *RON* exon 11 inclusion (in percent spliced-in, PSI) across TCGA samples do not depend on the average expression levels across samples **(a)** nor on the associated standard deviations **(b)**.

(c) Correlation between RBP expression and *RON* exon 11 inclusion in TCGA tumour samples partially recapitulates the observed effect of those RBPs in a previous knockdown (KD) screen[2]. Scatterplot compares Spearman correlation coefficients from TCGA samples with published *z*-scores (inverted sign) upon RBP KD. *HNRNPH2* is highlighted. *r*, Pearson correlation coefficient and corresponding *P*-value.

(d) *RON* exon 11 inclusion inversely correlates with *HNRNPH2* expression across 2,743 samples derived from 24 different healthy human tissues. Density scatterplot shows *HNRNPH2* expression (in TPM) and *RON* exon 11 inclusion (in PSI) across healthy samples from the Genotype-Tissue Expression (GTEx) project. *r*, Spearman correlation coefficient and corresponding *P*-value.

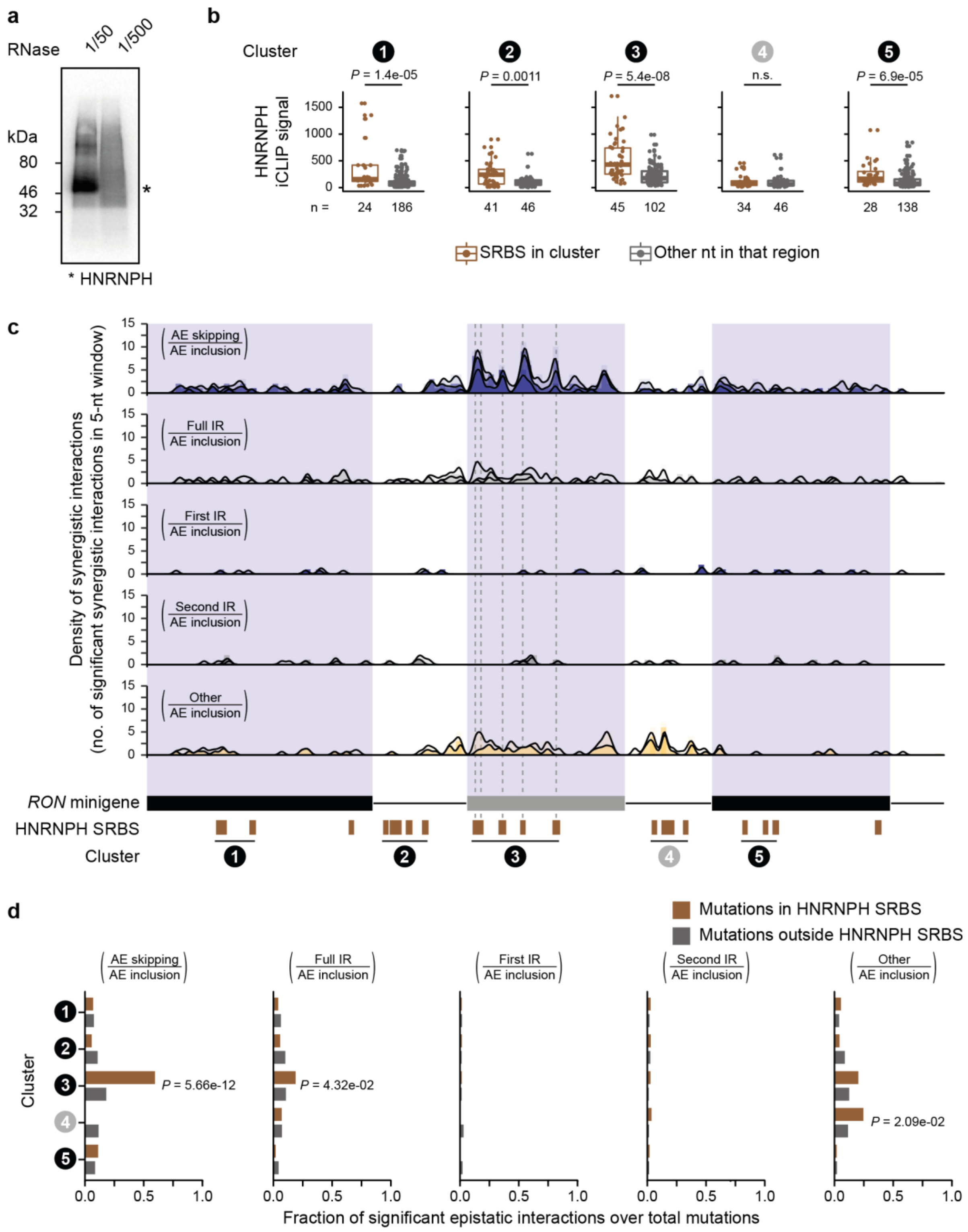

Supplementary Figure 12: HNRNPH iCLIP and synergistic interactions reveal functional HNRNPH binding sites. Related to Figs 4a and 5c.

(a) Autoradiograph shows crosslinked HNRNPH/RNA complexes that were treated with increasing RNase I dilutions prior to immunoprecipitation for optimisation of partial RNase digestion. Protein-RNA complexes run above expected molecular weight of HNRNPH (53 kDa; labelled by asterisk).

(b) HNRNPH crosslink events are significantly enriched in four out of five clusters of HNRNPH splice-regulatory binding sites (SRBS). Boxplots summarise HNRNPH iCLIP crosslink events on all nucleotides (nt) within SRBS ± 2 nt (brown) of each cluster (labelled by numbered circles) compared to all other positions within same exon/intron (grey). Number of positions in each box indicated below. Centre line and bounds of each box denote 25th, 50th and 75th percentile, and whiskers extend to most extreme values within 1.5x interquartile range (IQR). *P*-values correspond to two-sided Wilcoxon Rank-Sum test.

(c) Synergistic interactions between point mutations and *HNRNPH* KD are predominantly observed for AE inclusion, AE skipping, and ‘other’ isoforms. Bar diagrams for each splice isoform ratio show number of significant synergistic interactions (FDR < 0.1%) in adjacent 5-nt windows. Lines indicate the density in a 5-nt sliding window. Each panel

shows an overlay of increasing *z*-score cutoffs (|*z*|>2 >3, and >5), identifying a total of 354, 222 and 66 significant synergistic interactions, respectively (**Supplementary Table 2**). Splice sites ± 2 nt were excluded from this analysis. *RON* minigene structure and predicted HNRNPH SRBS clusters are given below.

(d) Synergistic interactions are significantly enriched within HNRNPH SRBS in cluster 3. Bar diagrams for each splice isoform ratio display the fraction of significant synergistic interactions over all mutations for SRBS within the five clusters (brown) compared to all other positions within same exon/intron (grey). Significant differences are shown with *P*-values correspond to one-sided Wilcoxon Rank-Sum test.

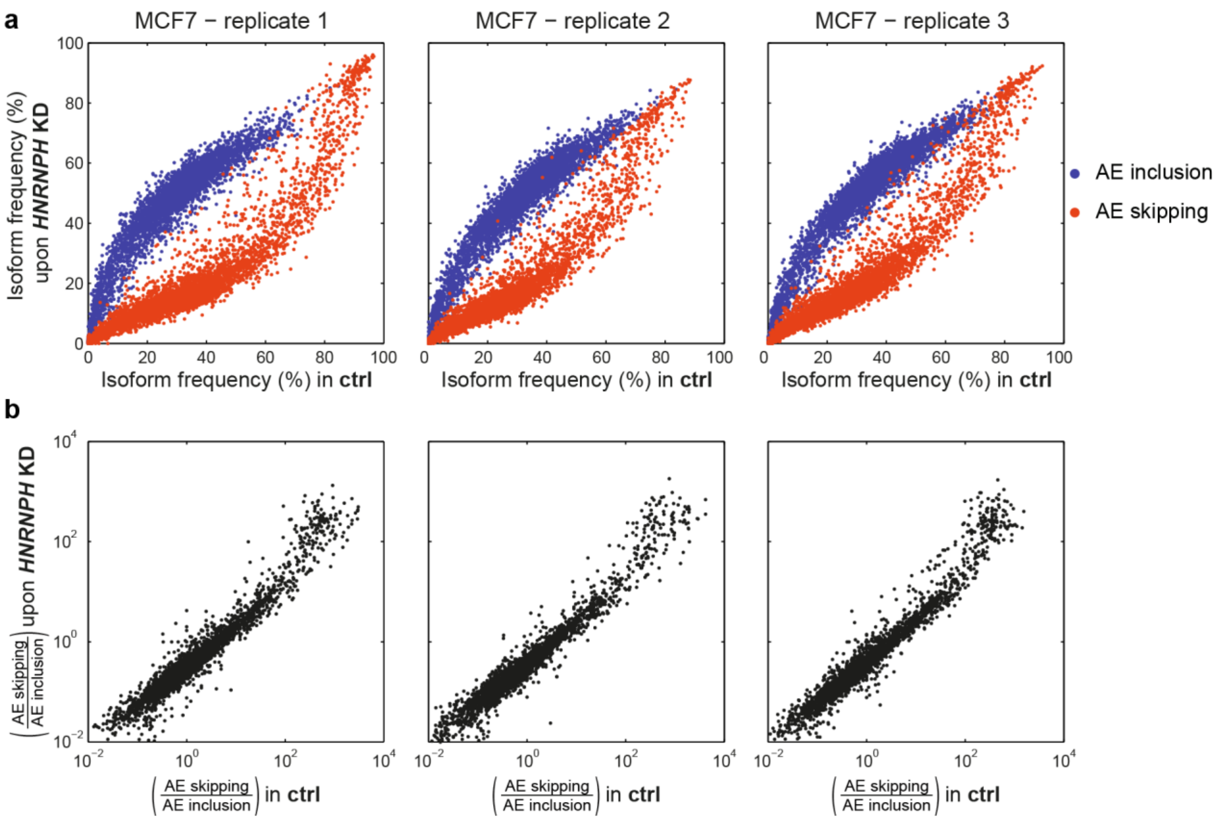

Supplementary Figure 13: *HNRNPH* KD shows non-linear effects on splice isoforms, while splice isoform ratios respond linearly.

(a) AE inclusion (blue) and AE skipping (red) isoform frequencies in MCF7 cells under control (ctrl) and *HNRNPH* KD conditions are shown for all individual minigene variants in three biological replicates. Depending on baseline frequency under control conditions, strength of KD-induced effect varies (top).

(b) Corresponding splice isoform ratios (AE skipping over AE inclusion) for individual minigene variants (black) are independent of baseline frequency and behave linearly.

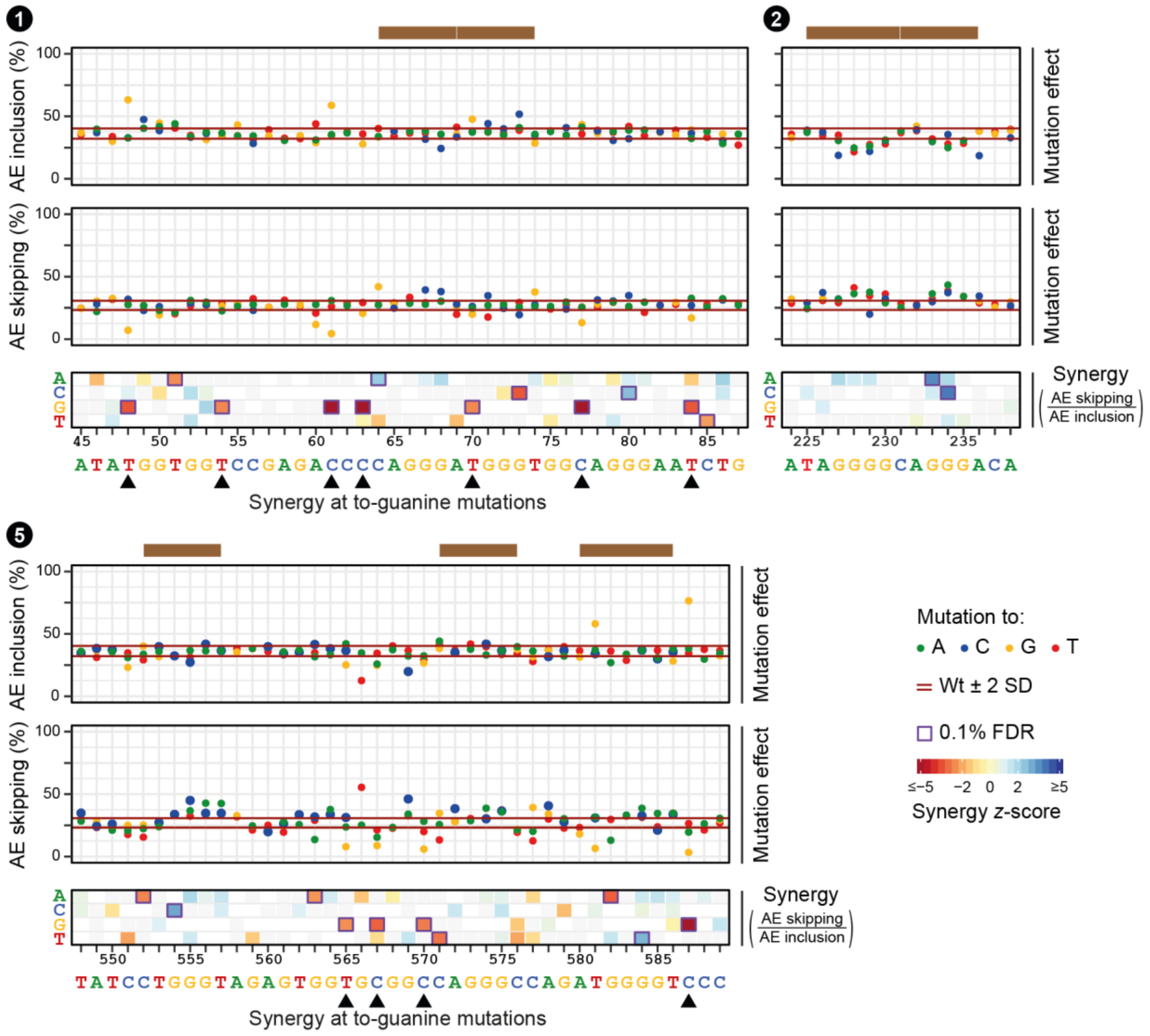

Supplementary Figure 14: Mutation effects and synergistic interactions between *HNRNPH* KD and single point mutations highlight mutations that reinforce HNRNPH binding. Related to Fig. 5d.

Within HNRNPH splice-regulatory binding sites (SRBS) of clusters 1 and 5 (indicated by numbered circles) in constitutive exons 10 and 12, respectively, mutations to guanines generally lead to increased AE inclusion, while AE skipping levels are reduced. Strong synergistic interactions of these mutations (highlighted by arrowheads) suggest that strengthening HNRNPH binding at these sites enhances its splicing-regulatory function. HNRNPH SRBS cluster 2 in first intron regulates AE skipping and AE inclusion in opposite direction compared to HNRNPH SRBS cluster 3 (**Fig. 5d**).

For each SRBS cluster, three plots are shown summarising single mutations effects on AE inclusion (top) and AE skipping (middle) as well as synergistic interactions of mutations with *HNRNPH* KD (based on splice isoform ratio of AE skipping over AE inclusion; bottom). Single mutation effects are displayed as dot plot, with y-axis showing the isoform frequency (mean of three biological replicates) resulting from each individual mutation in a given position along the y-axis. Each dot represents one mutation, with colours indicating inserted nucleotide (green, mutation to A; blue, to C; yellow, to G; red, to T). Red lines indicate median isoform frequency of wt minigenes ± 2 standard deviations (SD). HNRNPH SRBS (brown) are given above. Synergistic interactions are displayed as a heatmap of *z*-scores (mean of three biological replicates) as a quantitative measure of synergy between indicated mutation and *HNRNPH* KD. Each row represents one type of inserted nucleotide (indicated on the left). White and grey fields indicated mutations that were either not present or filtered out due to inconsistent signs (see Methods). Purple boxes highlight significant synergistic interactions (0.1% FDR).

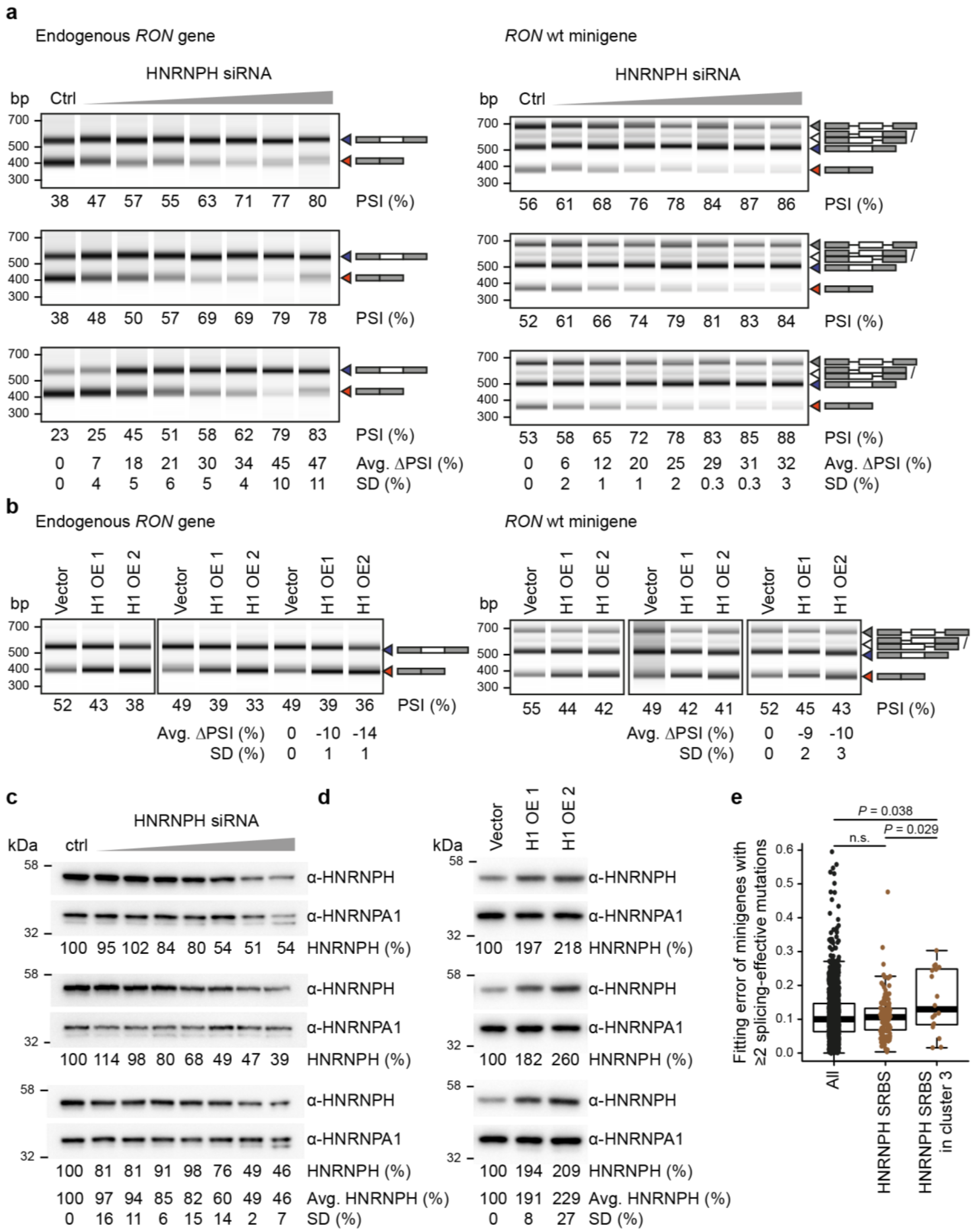

Supplementary Figure 15: *RON* exon 11 splicing is sensitive to reduced HNRNPH levels. Related to Fig. 6b.

(a) *RON* exon 11 inclusion for endogenous *RON* gene and wt *RON* minigene upon gradual reduction of HNRNPH using increasing concentrations of HNRNPH-specific siRNA. Semiquantitative RT-PCR results in MCF7 cells are visualised in a gel-like representation of capillary electrophoresis. Splice products are indicated on the right. Percent spliced-in (PSI) for each condition is given below. Average (Avg.) and standard deviation (SD) of splicing change (ΔPSI against non-targeting control siRNA, Ctrl) across the three replicates are given below. Whole gel images for these experiments are shown in Supplementary Fig. 16.

(b) *RON* exon 11 inclusion for the endogenous *RON* gene and the wt *RON* minigene upon gradual overexpression of *HNRNPH1* (H1 OE1/OE2) compared to a transfection with an empty vector control (Vector). Semiquantitative RT-PCR results of three biological replicates in MCF7 cells. Visualisation as in (a). Whole gel images for these experiments are shown in Supplementary Fig. 16.

(c) Western Blot analysis to quantify amount of HNRNPH upon gradual *HNRNPH* knockdown using increasing concentrations of HNRNPH-specific siRNA in three biological replicates. HNRNPA1 served as loading control. Relative HNRNPH abundance normalised against HNRNPA1 (in %) is given below. Average (Avg.) and standard deviation (SD) of HNRNPH abundance relative to non-targeting control siRNA (Ctrl) across the three replicates are given below. Whole gel images for these experiments are shown in Supplementary Fig. 16.

(d) Western Blot analysis to quantify amount of HNRNPH upon gradual *HNRNPH1* overexpression (H1 OE1/OE2) compared to empty vector transfection (Vector) in three biological replicates. Loading control and visualisation as in (c). Whole gel images for these experiments are shown in Supplementary Fig. 16.

(e) Minigenes with combination of splicing-effective mutations in HNRNPH SRBS cluster 3 show increased fitting errors, evidencing cooperative HNRNPH binding. Fitting error of minigenes with multiple splicing-effective mutations in HNRNPH SRBS cluster 3 is larger than for other minigenes containing splicing-effective mutations within other HNRNPH SRBS or elsewhere in the *RON* minigene. *P*-values correspond to one-sided Student’s t-test. Whole gel images for these experiments are shown in Supplementary Fig. 16.

endogenous *RON* gene

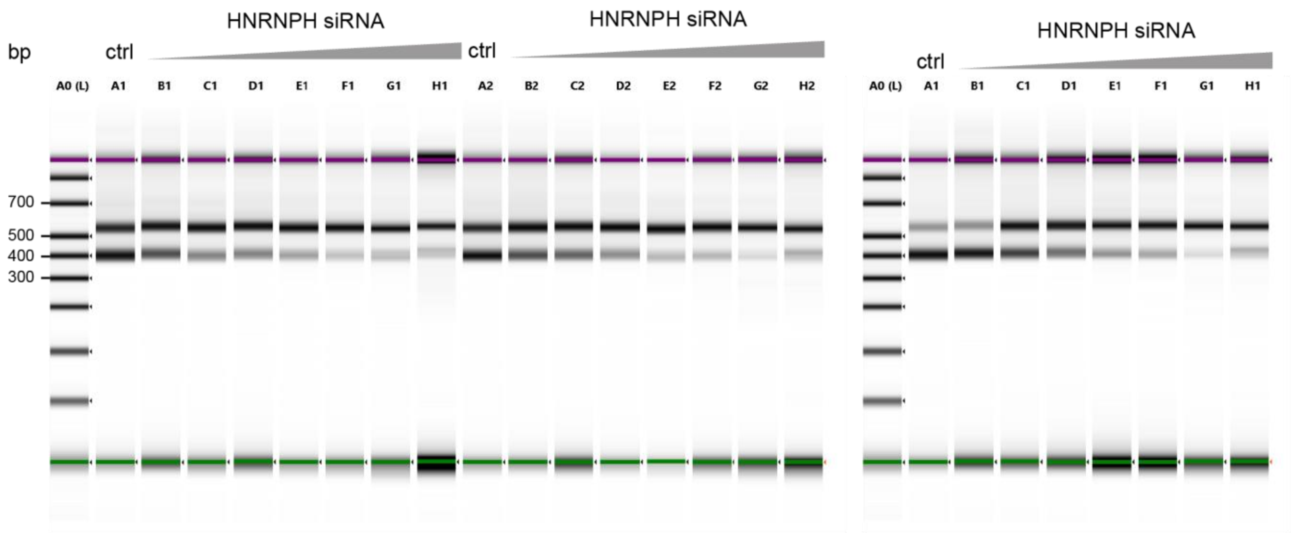

***RON* wt minigene**

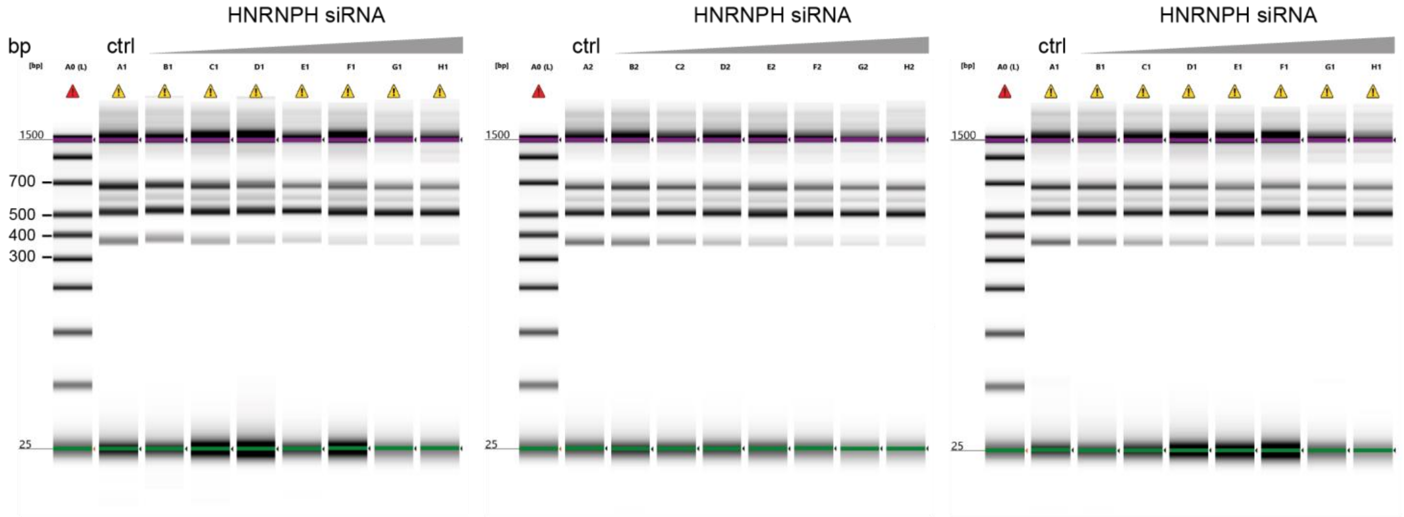

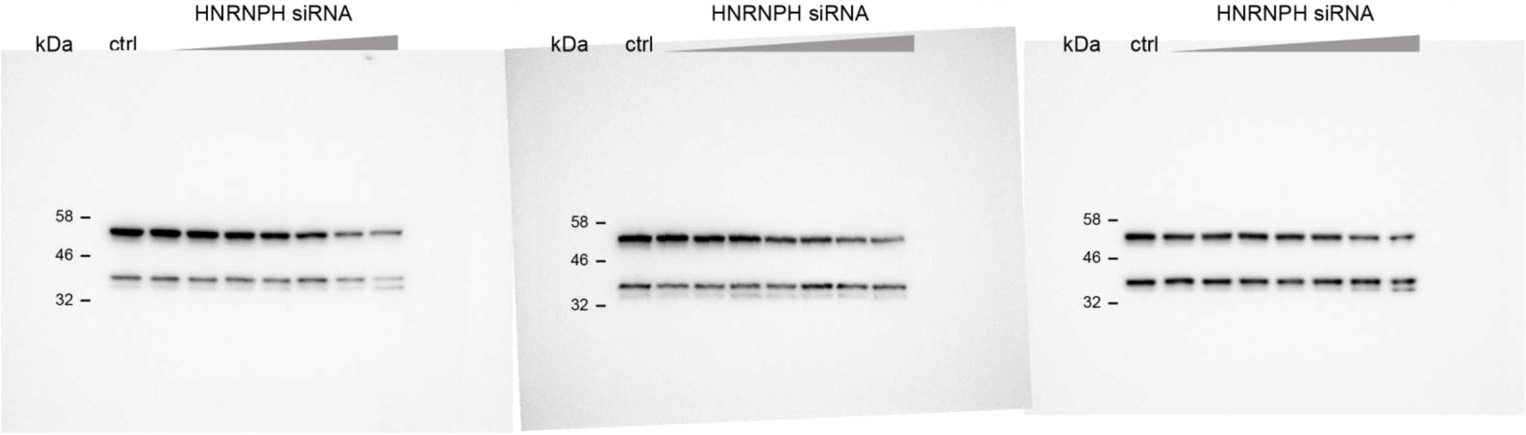

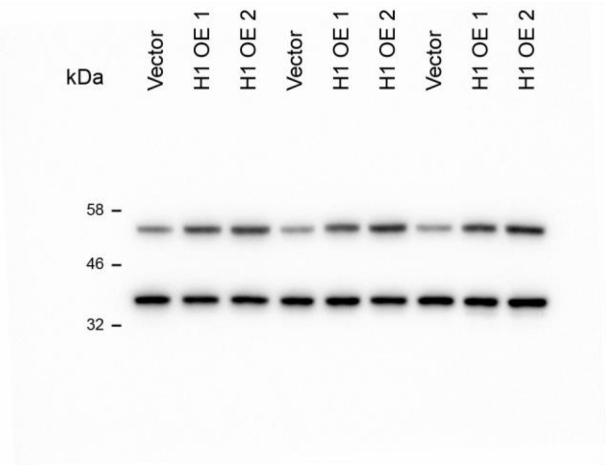

Supplementary Figure 16: Whole gel images for experiments shown in Supplementary Figure 15.

Semiquantitative RT-PCR analyses of *RON* exon 11 splicing upon gradual reduction of HNRNPH visualised in a gel-like representation of capillary electrophoresis (top) and corresponding Western blot analyses (bottom).

Supplementary References

1. Raue, A. *et al*. Structural and practical identifiability analysis of partially observed dynamical models by exploiting the profile likelihood. *Bioinformatics* **25**, 1923-1929 (2009).

2. Papasaikas, P., Tejedor, J. R., Vigevani, L. & Valcárcel, J. Functional splicing network reveals extensive regulatory potential of the core spliceosomal machinery. *Mol. Cell* **57**, 7-22 (2015).

3. Bonomi, S. *et al*. HnRNP A1 controls a splicing regulatory circuit promoting mesenchymal-to-epithelial transition. *Nucleic Acids Res.* **41**, 8665-8679 (2013).
