# Supplementary material for: Decoding a cancer-relevant splicing decision in the RON proto-oncogene using high-throughput mutagenesis
Source: Nat Commun. 2018 Aug 17;9:3315. doi: 10.1038/s41467-018-05748-7 (PMC6098099; doi:10.1038/s41467-018-05748-7)
Supplement: Supplementary file 3 — Description of Additional Supplementary Files [file 41467_2018_5748_MOESM3_ESM.pdf]

Description of Additional Supplementary Files

File Name: Supplementary Data 1
Description: Quantification of splicing isoform frequencies for all 5,791 minigene variants in the library. For each minigene variant, an assigned identification number (ID) is shown together with the 15-nt barcode sequence and the contained mutations, with multiple mutations separated by commas. Relative isoform frequencies of alternative exon (AE) inclusion, AE skipping, full intron retention (IR), first IR, second IR and other isoforms are given for RNA-seq replicates (rep) 1-3 from untreated HEK293T cells as well as control (ctrl) and *HNRNPH* KD MCF7 cells. Notation T216A, i.e. T in position 216 mutated to A; WT, wild type minigene without mutations; NA, minigene was either not present in the respective dataset or filtered out due to low read numbers (see Methods).

File Name: Supplementary Data 2
Description: Splicing measurements associated with point mutations in all positions along the *RON* minigene. Boxplot showing the frequencies of the alternative exon (AE) inclusion isoform (in %) measured by RNA-seq in HEK293T cells for all minigene harbouring a point mutation in a given minigene position (indicated on the x-axis). Each dot represents the measurement for an individual minigene variant, with colours indicating the inserted nucleotide (green, mutation to A; blue, to C; yellow, to G; red, to T). Blue and dashed purple lines represent interquartile range of all mutated and wt minigenes, respectively. Sequence of the wt *RON* minigene is given below.

File Name: Supplementary Data 3
Description: Single mutation effects and synergistic interactions between *HNRNPH* knockdown and point mutations inferred by the mathematical splicing model. The table provides the model estimates of splice isoform frequencies (in %) and synergistic interactions (as z-scores) in response to individual mutations (single nucleotide variants, SNV; insertions, IN; deletions, DEL) in HEK293T cells as well as MCF7 cells under control and *HNRNPH* knockdown (KD) conditions. Isoforms include alternative exon (AE) inclusion, AE skipping, full intron retention (IR), first IR, second IR, and other isoforms. For each sample type, separate worksheets are given for individual replicates (rep1-3) as well as their average ('average frequency'). Mean and standard deviation of isoform frequencies in the wt minigenes are given above (for calculating z-scores, see Supplementary Note 3). For MCF7 cells, the table also lists z-scores as a quantitative measure of synergistic interactions between the indicated mutation and the *HNRNPH* KD, with separate worksheets for individual replicates as well as their average ('average synergy'). In addition to position within *RON* minigene (notation A26T, i.e. A in position 26 mutated to T), genomic coordinates (human genome version GRCh37/hg19) are given for each mutation. Two additional worksheets contain median-based estimates for HEK293T and MCF7 (average over three biological replicates) for mutations that could not be inferred by the linear regression model as they result in high amounts of non-canonical isoforms ('other'; see Supplementary Note 1).

File Name: Supplementary Data 4
Description: Single mutation effects on all isoforms in HEK293T cells. For each isoform, the y-axis shows the isoform frequency (mean of three biological replicates) resulting from each individual mutation in a given position along the y-axis. Each dot represents one mutation, with colours indicating the inserted nucleotide (green, mutation to A; blue, to C; yellow, to G; red, to T). Red lines indicate the median isoform frequency of the wt minigenes (dashed line) ± 2 standard deviations (solid lines). The shown isoforms are alternative exon (AE) inclusion, AE skipping, full intron retention (IR), first IR, second IR and other isoforms. A schematic of the *RON* minigene together with HNRNPH crosslink events from iCLIP data (dark blue) and HNRNPH splice-regulatory binding sites (SRBS; brown) are given above.

File Name: Supplementary Data 5
Description: Mutation effects on *RON* exon 11 splicing in cancer patients. Related to Fig. 3c,d. Information on 51 mutations that are present in tumours but not matched normal samples from 153 patients in The Cancer Genome Atlas (TCGA), including the mutation, its genomic coordinate (human genome version hg19), the tumour cohort of the patient with the total number of patients and of mutation-bearing patients therein, the number of RNA-seq reads supporting the PSI in the TCGA samples (average across mutation-bearing samples from the cohort), as well as changes in alternative exon (AE) skipping from TCGA (in 1-PSI) and our screen (in % isoform frequency). Splicing effects from TCGA and our screen are only given for mutations from cohorts with more than 24 supporting reads on average (highlighted in blue; used in Fig. 3c). Abbreviations of cancer types: BLCA, Bladder Urothelial Carcinoma; BRCA, Breast Invasive Carcinoma; CESC, Cervical Squamous Cell Carcinoma and Endocervical Adenocarcinoma; COAD, Colon Adenocarcinoma; DLBC, Lymphoid Neoplasm Diffuse Large B-cell Lymphoma; ESCA, Esophageal Carcinoma; HNSC, Head-Neck Squamous Cell Carcinoma; KIRC, Kidney Renal Clear Cell Carcinoma; KIRP, Kidney Renal Papillary Cell Carcinoma; LIHC, Liver Hepatocellular Carcinoma; LUAD, Lung Adenocarcinoma; LUSC, Lung Squamous Cell Carcinoma; OV, Ovarian Serous Cystadenocarcinoma; PAAD, Pancreatic Adenocarcinoma; SKCM, Skin Cutaneous Melanoma; STAD, Stomach Adenocarcinoma; THCA, Thyroid Carcinoma; THYM, Thymoma; UCEC, Uterine Corpus Endometrial Carcinoma.

File Name: Supplementary Data 6
Description: Single mutation effects and synergistic interactions with *HNRNPH* knockdown for all isoforms in MCF7 cells. For each isoform, two plots are shown summarising single mutations effects (top) and synergistic interactions (bottom). The shown isoforms are alternative exon (AE) inclusion, AE skipping, full intron retention (IR), first IR, second IR and other isoforms. Note that the synergistic interactions are calculated from ratios of a given isoform over AE inclusion, so not synergistic interactions are shown for AE inclusion. Top for each isoform: Single mutation effects are displayed as dot plot, with the y-axis showing the isoform frequency (mean of three biological replicates) resulting from each individual mutation in a given position along the y-axis. Each dot represents one mutation, with colours indicating the inserted nucleotide (green, mutation to A; blue, to C; yellow, to G; red, to T). Red lines indicate the median isoform frequency of the wt minigenes (dashed line) ± 2 standard deviations (solid lines). A schematic of the *RON* minigene together with HNRNPH crosslink events from iCLIP data (dark blue) and HNRNPH splice-regulatory binding sites (SRBS; brown) are given above. Bottom for each isoform: Synergistic interactions are displayed as a heatmap of z-scores (mean of three biological replicates) as a quantitative measure of synergy between the

indicated mutation and the *HNRNPH* KD. Each row represents one type of inserted nucleotide (indicated on the left). White and grey fields indicated mutations that were either not present or filtered out due to inconsistent signs (see Methods). Purple boxes highlight significant synergistic interactions (0.1% FDR).

File Name: Supplementary Data 7
Description: Correlation of RNA-binding protein expression with *RON* exon 11 splicing in TCGA tumour samples and healthy tissues. Related to Figs 3f, 6c, and Supplementary Fig. 11. The Spearman correlation coefficients of transcript expression levels of 190 RNA-binding proteins (RBPs) across 4,514 tumour samples (TCGA) or 2,743 normal samples (GTEx) with *RON* exon 11 splicing are shown along with significance of the correlations (*p*-value) and false discovery rate (FDR). Regression slope correspond to linear regression between RBP expression and *RON* exon 11 percent spliced-in (PSI) in TCGA tumour samples. Source indicates whether a candidate RBP was identified by ATtRACT analysis (this study) or a previous publication (Papasaikas et al., Mol Cell, 2015, 57(1):7-22).

File Name: Supplementary Data 8
Description: Splicing quantifications by semiquantitative RT-PCR upon transfections of individual minigene variants. Related to Figs 1e, 2d, 5e and Supplementary Fig. 4b. (i) 59 minigene variants that were randomly picked from the library, validated by Sanger sequencing and characterised in HEK293T cells using semiquantitative RT-PCR (Fig. 1e). Since these minigenes were generated as part of the minigene library, they contain a 15-nt barcode sequence and variable numbers of mutations. Values correspond to isoform frequencies (in %). (ii) 26 minigene variants to assess the accuracy of the model predictions for mutations with different occurrence in the library (Fig. 2d and Supplementary Fig. 4b). Values correspond to changes in isoform frequencies compared to wt *RON* minigenes (delta; in %) in HEK293T cells. (iii) 10 minigenes with targeted mutations in HNRNPH SRBS from all five initially predicted clusters that were measured together with the wt *RON* minigene under control and *HNRNPH* knockdown (KD) conditions in MCF7 cells (Fig. 5e). Since the first IR and second IR isoforms cannot be discriminated in RT-PCR, they were summed up into 'partial IR'. Quantifications for (ii) and (iii) are averages of three biological replicates.
